# Drying characteristics, environmental and economic analysis of a solar dryer with evacuated tube solar collector for drying Nile Tilapia slices

**DOI:** 10.1038/s41598-025-94194-9

**Published:** 2025-03-21

**Authors:** Omar Shahat Younis, Awad Ali Tayoush Oraiath, Khaled A. Metwally, Eldessoky S. Dessoky, Samy F. Mahmoud, Mohamed Nageeb Rashed, Aml Abubakr Tantawy, Said Elshahat Abdallah, Mohamed Hamdy Eid, Mohamed Farag Taha, Omar Saeed, Abdallah Elshawadfy Elwakeel

**Affiliations:** 1 Food Manufacturing Engineering and Packaging Department, Food Technology Research Institute, Agriculture Research Center, Giza, Egypt; 2 Department of Agricultural Engineering, Faculty of Agriculture, Omar Al Mukhtar University, P.O. Box 991, Al Bayda, Libya; 3 Soil and Water Sciences Department, Faculty of Technology and Development, Zagazig University, Zagazig, 44519 Egypt; 4 Department of Biology, College of Science, Taif University, Taif, Saudi Arabia; 5 Department of Biotechnology, College of Science, Taif University, Taif City, Saudi Arabia; 6 Chemistry Department, Faculty of Science, Aswan University, Aswan, Egypt; 7 Food Science and Technology Department, Faculty of Agriculture and Natural Resources, Aswan University, Aswan, Egypt; 8 Agricultural Engineering Department, Faculty of Agriculture, Kafrelsheikh University, Kafr El Sheikh, Egypt; 9 Institute of Environmental Management, Faculty of Earth Science, University of Miskolc, Miskolc-Egyetemváros, 3515 Hungary; 10 Geology Department, Faculty of Science, Beni-Suef University, Beni-Suef, 65211 Egypt; 11 School of Agricultural Engineering, Jiangsu University, Zhenjiang, 212013 China; 12 Department of Soil and Water Sciences, Faculty of Environmental Agricultural Sciences, Arish University, Arish, North Sinai 45516 Egypt; 13 Doctoral School of Environmental Science, Hungarian University of Agriculture and Life Sciences (MATE), Páter Károly U. 1, Gödöllő, 2100 Hungary; 14 Agricultural Engineering Department, Faculty of Agriculture and Natural Resources, Aswan University, Aswan, Egypt

**Keywords:** Environmental analysis, Fish drying, Sustainable agricultural systems, Carbon footprint, Oven drying, Ecology, Environmental sciences

## Abstract

Lake Nasser in Egypt contains significant tilapia fish quantities, yet consumption remains low due to its geographical isolation from marketing and consuming areas. Therefore, investigating efficient and economical Tilapia fish drying methods is essential. The current study developed and tested a solar dryer based on solar energy collection, using evacuated tubes at three Nile Tilapia slice (NTS) thicknesses of 4, 8, and 12 mm, and an air velocity of 0.5 m/s. The obtained result of the solar dryer with evacuated tubes (SDET) was compared with the other results of the oven liquid petroleum gas (OLPG) as an industrial drying method. The results obtained showed that the air temperature inside the drying room of the SDET ranged between 44 and 75 °C. The average initial moisture content (MC) was 74.83% (w.b.). For both systems, the drying time ranged between 13 and 17 h at the same slice thickness. The effective moisture diffusivity was in the range of 0.87 × 10^–11^ to 5.66 × 10^–11^ m^2^/s. Furthermore, the mathematical modeling revealed the Modified Midilli (II) and Modified Henderson and Pabis models as the most suitable models to describe the drying behavior of NTS dried on SDET. On the other hand, the environmental analysis indicates that the developed SDET can mitigate approximately 273.6 tons of CO_2_ during its lifetime, resulting in a carbon credit equivalent of approximately 19,838.89 $. Additionally, the economic analysis of the SDET showed that the annual production of dried fish was 450 kg; this may result in substantial cost savings, amounting to a total of 608.4 $ per year. Also, the developed SDET had a payback period of approximately 0.413 years or less than half a year.

## Introduction

Fish is one of the most affordable sources of animal protein for many cultures, particularly for those who avoid red meat, as well as for malnourished individuals, immunocompromised patients, pregnant women, and nursing mothers. For centuries, various fish species have been a staple in the diets of ethnic groups across all continents. While fishing plays a major role in supplying fish meat, the growth of commercial aquaculture remains limited. Due to its high protein content, fish is often used as a key ingredient in meals. Additionally, fish holds substantial economic value, with fisheries and aquaculture providing employment, recreation, trade, and economic benefits to those involved in the industry. Fishery products constitute a significant segment of international trade, currently valued at over USD 50 billion^1^. Nile tilapia (*Oreochromis niloticus*), although native to African freshwater ecosystems, have been introduced to numerous nations both inside and beyond Africa. The introduction of Nile tilapia commenced in the latter half of the twentieth century, particularly in Southeast Asia and the Americas, primarily for aquaculture and fisheries improvement. Many of these introductions were firmly established in aquaculture. Consequently, the aquaculture of Nile Tilapia has been consistently increasing in several nations. These fish are vital to the livelihoods of local communities across various continents^2,3^. It proliferates swiftly, reproduces effortlessly, and acclimatizes to diverse environmental circumstances^4^. Nile tilapia is widely accepted and utilized in various cuisines due to its mild flavor and favorable texture, as any religious observance does not restrict it^4,5^. It consists of up to 80% water and is therefore highly perishable^6^. Thus, processing and preservation are essential for prolonging shelf life. An effective approach for preserving agricultural items such as fish is drying them using well-engineered machinery that optimizes energy use. In the absence of drying procedure, free water leads to the deterioration of food items^7,8^. Consequently, extracting the moisture from the fish fillet extends the shelf life of the dried fish fillet and facilitates packing and transportation^9,10^. The traditional approach of open-sun drying is facing contemporary challenges arising from the widespread adoption of energy-intensive methods and the quality of drying. In response, solar dryers have emerged as a sustainable alternative, utilizing solar thermal energy to effectively dehydrate vegetables^11,12^.

Research indicates that global energy usage doubles every 20 years^13^. Currently, renewable energy use remains significantly lower than that of fossil fuels, leading to widespread environmental issues and pollution^14^. However, in recent decades, there has been increasing interest in adopting renewable and sustainable energy sources, particularly solar energy^15,16^. In response to increasing energy demands, efforts have been made to utilize non-conventional energy sources for efficient clean energy generation. Solar energy has consistently garnered popularity among humans. Solar energy has become increasingly popular for its potential in heating applications^17–19^. Solar drying has emerged as a promising technique for fish processing worldwide^20^, offering one of the most efficient methods for fish desiccation through both traditional and industrial processes^21,22^. However, traditional sun drying in open environments produces lower quality and less valuable products compared to industrial methods^23^. The simple design of solar dryers and the widespread availability of solar energy have contributed to their global adoption^24^. Despite the advantages of solar drying, such as, cost savings and accessibility—progress has been limited due to the relatively low efficiency of solar dryers. As a result, various efforts have been made to improve their performance^25–27^.

The solar dryer market size was over USD 3.16 billion in 2024 and is anticipated to cross USD 6.58 billion by 2037, growing at more than 5.8% CAGR during the forecast period, i.e., between 2025 and 2037. In the year 2025, the industry size of solar dryer is estimated at USD 3.32 billion^28^. Solar dryers exhibit many designs; nevertheless, they generally comprise two primary components: the solar collector and the drying chamber^29,30^. A solar collector is engineered to capture and transform solar radiation into thermal energy. In a solar dryer, many types of collectors are typically integrated with the dryer, such as flat plate solar collectors^31^, solar air collectors with phase change material (PCM)^32^, evacuated tube solar collectors, thermal storage system and PCM container inside^7^, vertical collector chimney^33^, solar air collectors provided with artificial square-shaped roughness^34^, phase change materials at different positions in the collector^35^, solar tracking collectors^36^, double-pass flat and v-corrugated plate solar air heaters^37^, single and double slope solar dryer^38–40^, flat plate solar collectors using a reflector^41^. Conversely, it is primarily classified into three categories: flat plate collector, evacuated tube collector, and concentrating collector^17,42–45^.

Also, there have been many attempts to increase the thermal efficiency (TE) of the SC. ElGamal^46^ developed an SD-integrated ASCT for drying apple fruit, and they reported that the TE of the SD reached 45% compared to the traditional SD. Bhowmik et al.^41^ improved the TE of a flat-plate SC using solar reflectors. The result showed that the TE was increased by about 10% compared to the traditional system. Zheng et al.^47^ numerically investigated a compound parabolic concentrator SC, and the results showed that the TE of the SC was 60.5%. Zou et al. a small parabolic-trough SC, where the TE of the SC reached about 67%. A heat-pipe evacuated tube with an SC was created by Chamsaard et al.^48^, and the findings revealed that the collector’s TE was 78%. Rittidech et al.^49^ found that the TE of the SC reached 76% when using a circular glass tube. Wei et al.^50,51^ evaluated a flat-plate heat SC with a heat pipe where the TE of the SC reached 66%. The operation of an individual spiral-shaped SC tube and the number of riser tubes linked to headers in a standard SC were compared by Verma et al.^51^, where the result showed that the TE increased by 21.94% under a forced mode compared with the traditional SC. Ramachandran et al.^52^ compared the performance of a flat-plate SC and a Scheffler solar concentrator, and the results indicated that the TE was increased by 6% when using a chaffier concentrator. Das et al.^53^ compared the performance of an SD integrated with an ASCT with the traditional system, and they reported that the TE was increased by up to 75.7% by using the tracking system. A biaxial solar tracker that is mirror-reflected was devised and assessed by Ismail et al.^54^. According to their study, the AT was 15% higher on average than that of the fixed panel.

Mathematical models are essential for analyzing drying kinetics, which is vital for regulating the drying process and is of considerable theoretical and practical significance in ensuring drying quality, minimizing energy consumption, and optimizing the drying procedure^55–57^. These models enable the application of knowledge acquired from food drying tests to industrial contexts^55,58^. Numerous mathematical models have been developed by various researchers for different materials, including those by Lewis (Newton), Page, Henderson, and Logarithmic^59^, which have been employed to forecast the drying process of agricultural products, particularly fruits^60^ and vegetables^61^. Many mathematical models have also been applied to aquatic products, such as the air drying process of Atlantic salmon^62^, the radio frequency combined hot air drying process of Tilapia skin^63^, three different drying processes of scallop adductor muscle^64^, the microwave drying of Jaya fish^65^, and the blast electric oven salted Otolithes Ruber^66^. The rising energy consumption, especially for drying aquatic items, necessitates urgent innovation in drying techniques. Where Lake Nasser in Egypt contains significant Tilapia fish quantities, yet consumption remains low due to its geographical isolation from marketing and consuming areas. Therefore, investigating efficient and economical tilapia fish drying methods is essential. Most research analyzing the drying performance of Nile tilapia has not investigated the mathematical modeling of the drying process using solar dryers integrated with evacuated tube solar collectors, nor have they examined the effective moisture diffusivity values. Moreover, there is a lack of knowledge regarding the environmental and economic analysis of the drying process for NTS. So, this work involved the development of an indirect solar dryer combined with an evacuated tube solar collector, followed by a performance comparison with an oven liquid petroleum gas (OLPG). The primary objectives of this study are: (i) to examine the technical and economic analysis of the developed SDET, (ii) to determine the effective moisture diffusivity (EMD) value of Nile tilapia slices for both drying systems to evaluate the thin layer drying behavior, and (iii) to conduct an environmental impact analysis of the developed SDET to assess the system’s effectiveness.

## Materials and methods

### Materials

**Fig. 1:**
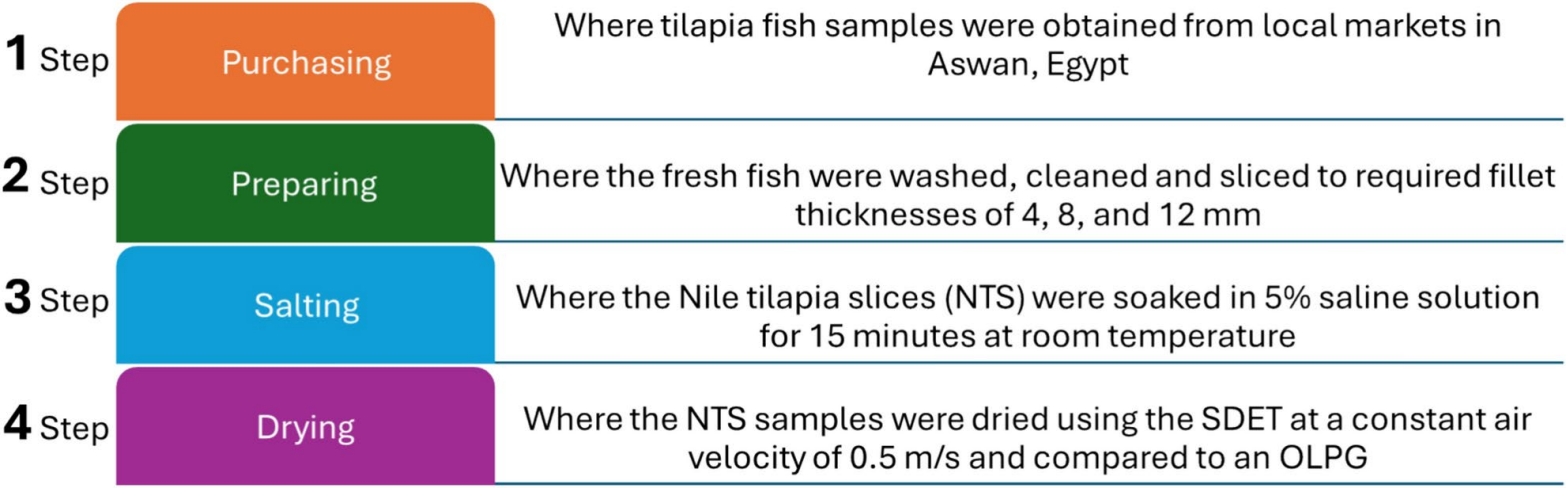
Preparation steps of the NTS.

**Fig. 2:**
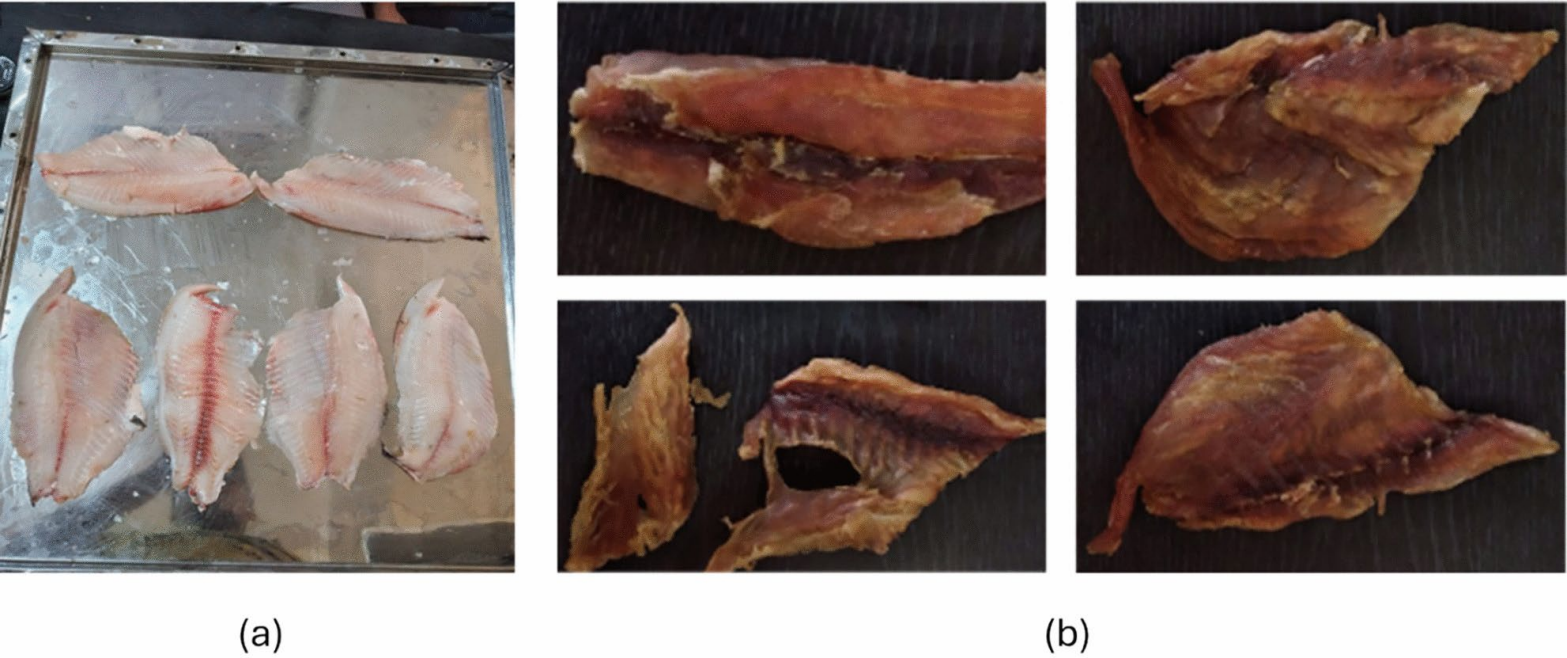
Nile tilapia fish fillet. Whereas (**a**) fresh and (**b**) dried.

All drying experiments were performed at the Agricultural Research Center in Giza, Egypt, in 2024. During the present study, tilapia fish samples were obtained from local markets in Aswan, Egypt. The preparation processes of the NTS are illustrated in Fig. 1, this figure was generated by Microsoft office 2016. The average initial moisture content of the NTS samples was determined to be 74.83% on a wet basis (w.b.) (297.3% on a dry basis (d.b.)), as assessed using a convection oven at 103 ± 1 °C^67^. Figure 2 shows fresh and dried Nile Tilapia fish samples; this figure was edited by Microsoft office 2016.

### Experimental set-up

**Fig. 3:**
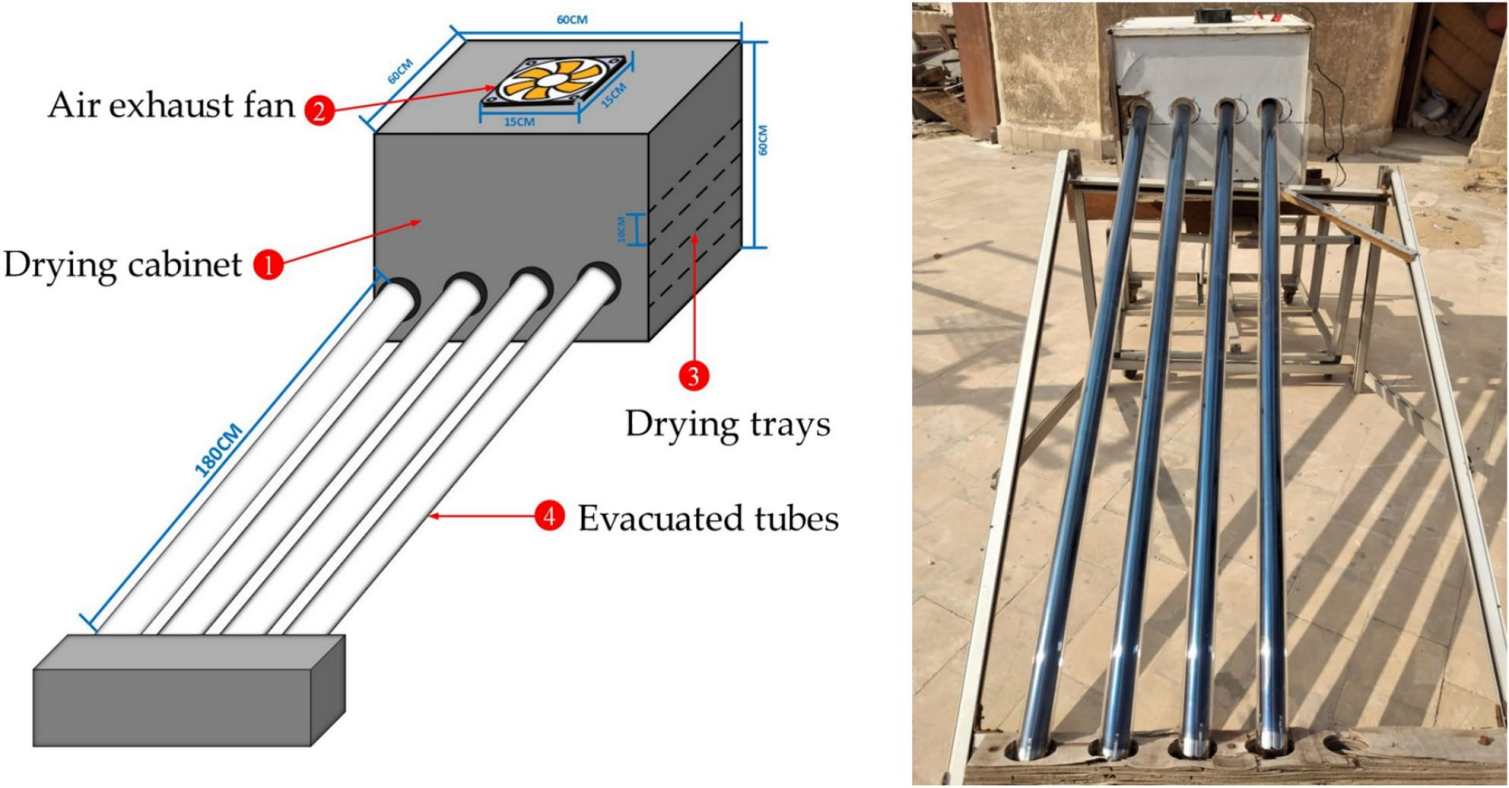
Schematic illustration of the SDET, (AutoCAD software 2020).

**Fig. 4:**
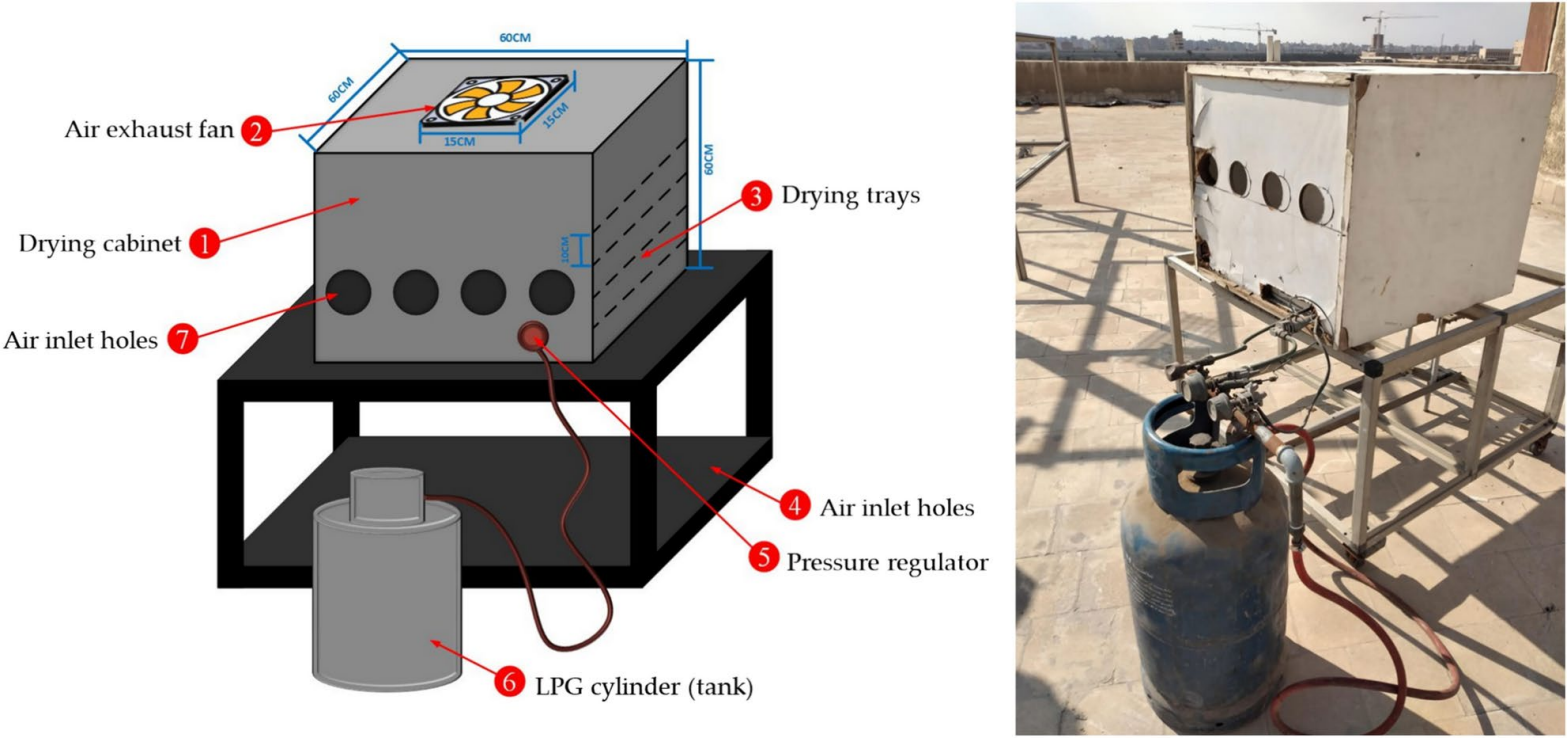
Schematic illustration of the OLPG, (AutoCAD software 2020).

**Fig. 5:**
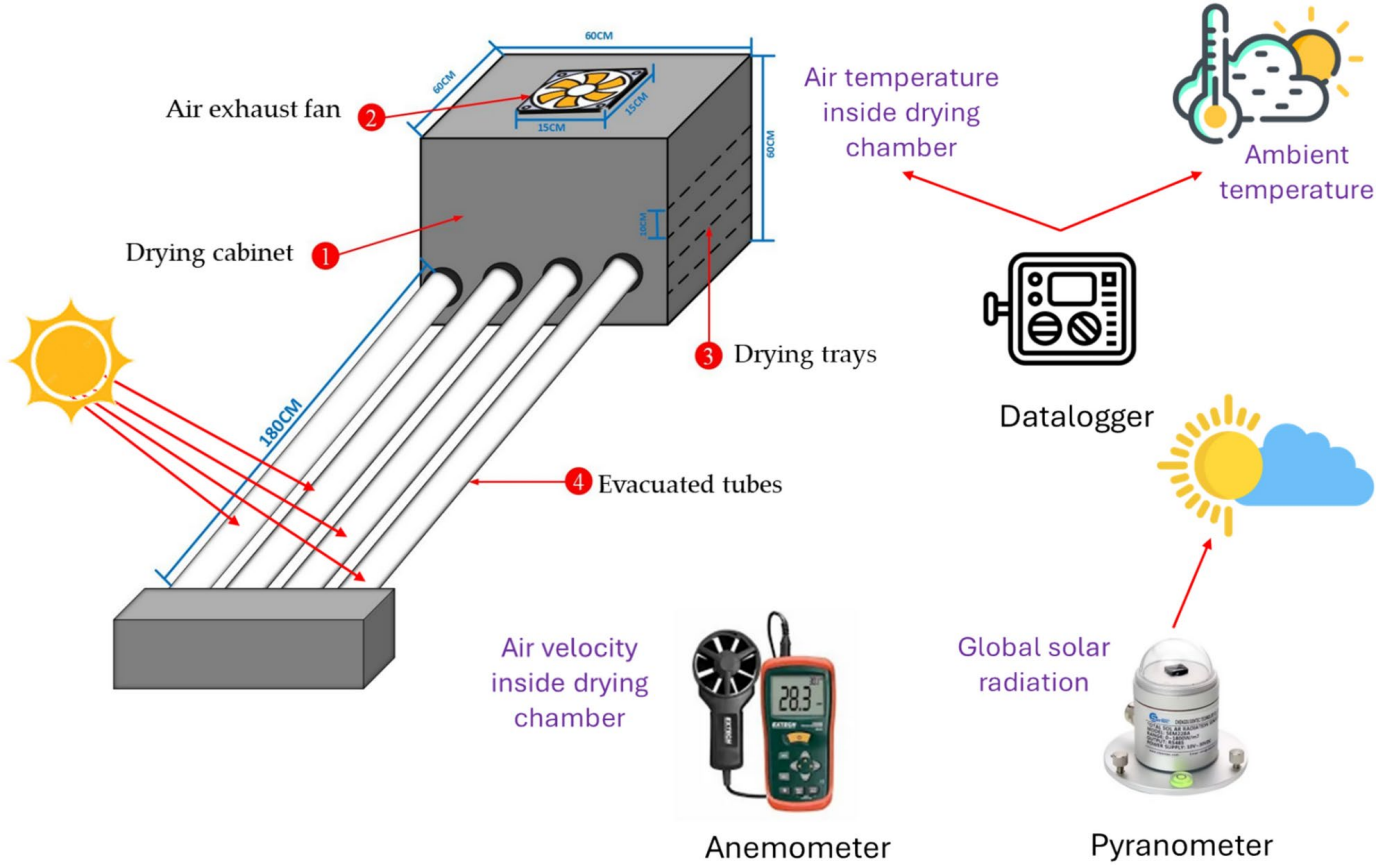
schematic illustration of the experimental setup connected with measuring instrumentation, (AutoCAD software 2020).

The diagrams of the SDET and OLPG are presented in Figs. 3 and 4. This study aims to experimentally assess the performance of the SDET, which incorporates an evacuated tube solar collector (ETSC). The ETSC comprises four borosilicate glass vacuum tubes (outer diameter = 58 mm, inner diameter = 47 mm) with a length of 1800 mm, accompanied by co-axial pipes (20 mm), as illustrated in Fig. 3. The evacuated tube in the experimental setup consists of two concentric glass tubes constructed from strong borosilicate glass. A vacuum of 5 × 10^−2^ Pa is established between the two concentric tubes to reduce convective losses from the inner to the outer tube. The inner tube is enveloped in a layer of Al-N/Al to optimize solar radiation absorption and minimize reflection. The evacuated tubes were positioned within a wooden frame at a 30° angle, and at the top, they were linked to a drying room of 600 mm × 600 mm × 600 mm. The header section comprised two parallel manifolds, M1 and M2, for airflow management. Each manifold features one closed end and the opposite end designated for inlet/outlet purposes. The voids and surfaces of these manifolds were adequately insulated with thermocol (polystyrene) and glass wool to avert heat loss. The air suction fan was positioned at the apex of the drying room. The air velocity was maintained at a constant speed of 0.5 m/s according to Basiouny^68^, with an accuracy of ± 0.1 m/s, as measured by an anemometer (model: Extech AN100, China). Lutron flowed at a right angle to the bed. The OLPG depicted in Fig. 4 served as a benchmark to evaluate the efficacy of the created SDET in this investigation, wherein fish samples of identical thickness were desiccated for the same duration. The OLPG maintained an internal temperature of 60 °C. The moisture loss of the samples was measured using a balance at one-hour intervals until no significant weight change was detected. Additionally, sun radiation was measured using solar radiation sensors (model:_SENTEC RS485, Sichuan, China), while relative humidity, ambient air temperature, and air temperature within the drying room were recorded hourly throughout the drying period using dry humidity and temperature sensor (model: DHT-22, China). The schematic with dimensions and a real photograph of the experimental setup is illustrated in Fig. 5. Where Figs. 3, 4 and 5 were generated by AutoCAD software 2020 (version 23.1) and edited by Microsoft office 2016.

### Uncertainty analysis

The uncertainties in measuring instruments are estimated using the following relation according to Suraparaju et al.^11^:

1 $${\mathcal{W}}_{r} = \left[ {\left( {\frac{\partial R}{{\partial x_{1} }}{\mathcal{W}}_{1} } \right)^{2} + \left( {\frac{\partial R}{{\partial x_{2} }}{\mathcal{W}}_{2} } \right)^{2} + \cdots + \left( {\frac{\partial R}{{\partial x_{3} }}{\mathcal{W}}_{3} } \right)^{2} } \right]^{1/2}$$

The solar drying process analysis initially assessed moisture reduction and drying rate uncertainty at 1.15%. This estimate accounted for measuring instrument errors. A thorough analysis of daily output showed that measurement deviations were within a precise range of ± 0.7%. The study also addressed temperature, humidity, and solar radiation measurement errors of 0.3%, 0.25%, and 0.08%. After considering these considerations, the overall assessment of solar dryer efficiency was projected to have a cumulative imprecision of about ± 2%. This indicates that despite the inherent measurement uncertainties, the solar drying system remains reliable and effective in its operation. Cumulative imprecision underscores the robustness of the system in real-world conditions.

### Modelling of the drying process

The MC of drying NTS sample at time (t) can be transformed to be moisture ratio (MR) using Eq. (2), as mentioned by Daevishi et al.^69^:

2 $$MR = \frac{{M_{t} - M_{e} }}{{M_{0} - M_{e} }}$$

where M_t_, M_0_ and M_e_ are moisture content at any time of drying (kg_water_/kg_dry matter_), initial and equilibrium MCs (kg_water_/kg_dry matter_), respectively.

Equation (2) was simplified to “$MR = \frac{{M_{t} }}{{M_{0} }}$” by certain researchers ^70,71^, because of the persistent variation in the relative humidity of the drying air during the solar drying process. The drying rate (DR) of NTS samples was calculated using Eq. (3), as reported by Metwally et al.^24^:

3 $$DR = \frac{{M_{t} - M_{{\left( {t + dt} \right)}} }}{{d_{t} }}$$

where M_(t+dt)_ is the moisture content at (t + dt) and t is drying time (h).

The drying data acquired were analyzed using nonlinear least squares regression to match eleven thin-layer drying models outlined in Table 1. Statistical analyses of the experimental data were conducted utilizing SPSS 17.0 and Microsoft Excel (2020).

**Table 1:** Selected mathematical modeling to demonstrate the NTS drying process.

No	Model name	Model equation*	References
1	Newton (Lewis)	$MR = exp\left( { - kt} \right)$	^72^
2	Page	$MR = exp\left( { - kt^{n} } \right)$	^72^
3	Combined Two-term and Page	$MR = a exp\left( { - kt^{n} } \right) + b exp\left( { - ht^{n} } \right)$	^72^
4	Modified Henderson and Pabis	$MR = a\exp \left( { - kt} \right) + b exp\left( { - gt} \right) + c exp\left( { - ht} \right)$	^72^
5	Modified Midilli II	$MR = a exp\left( { - kt^{n} } \right) + b$	^72^
6	Modified Page III	$MR = k {\text{exp}}\left( { - \frac{t}{{d^{2} }}} \right)^{n}$	^72^
7	Modified Two Term III	$MR = a {\text{exp}}\left( { - kt} \right) + \left( {1 - a} \right) exp\left( { - kat} \right)$	^72^
8	Logistics	$MR = \frac{b}{{1 + a\exp \left( {kt} \right) }}$	^72^
9	Simplified Ficks Diffusion	$MR = a\exp \left( { - c\left( {\frac{t}{{L^{2} }}} \right)} \right)$	^73^
10	Approximation diffusion	$MR = a\exp \left( { - kt} \right) + \left( {1 - a} \right)exp\left( { - kbt} \right)$	^72^
11	Parabolic model	$MR = a + bt + ct^{2}$	^72^

*MR is the moisture ratio, dimensionless; *k* is the drying constant*,* h^−1^; t is the drying time, h; *L* is the thickness of the samples (slab), m; *a, b, c, d, g, h*, and *n* are the models constants, dimensionless.

The coefficient of determination (R^2^) is a fundamental criterion for selecting the optimal model to characterize the drying curves. Alongside R^2^, reduced chi-square (*χ*^2^), root mean square error (RMSE), sum square error (SSE), and average percentage error (E, %) are employed to assess the quality of the fit. The optimal model for characterizing the drying properties of samples was selected based on the highest coefficient of determination, the lowest reduced chi-square, root mean square error, and mean relative percent error^74,75^. The following method can be employed to calculate these parameters:

4 $$R^{2} = 1 - \frac{{\mathop \sum \nolimits_{i = 1}^{N} (MR_{pre, i} - MR_{exp, i} )^{2} }}{{\mathop \sum \nolimits_{i = 1}^{N} (\overline{M}R_{pre} - MR_{exp, i} )^{2} }}$$

5 $$\chi^{2} = \frac{{\mathop \sum \nolimits_{i = 1}^{N} (MR_{pre, i} - MR_{exp, i} )^{2} }}{N - n}$$

6 $$RMSE = \sqrt {\frac{1}{N}\mathop \sum \limits_{i = 1}^{N} (MR_{pre, i} - MR_{exp, i} )^{2} }$$

7 $$SSE = \frac{1}{N}\mathop \sum \limits_{i = 1}^{N} (MR_{pre, i} - MR_{exp, i} )^{2}$$

8 $$E\left( \% \right) = \frac{100}{N}\mathop \sum \limits_{i = 1}^{N} \left| {\frac{{MR_{exp, i} - MR_{pre, i} }}{{MR_{exp, i} }}} \right|$$

where MR_exp,i_ is experimental MR; MR_pre,i_ is predicted MR; N is number of observations; n is number of constants.

### Effective moisture diffusivity (EMD)

In the majority of studies on drying, diffusion is widely recognized as the primary process for the transport of moisture to the surface for evaporation. The EMD can be calculated from the slope of the normalized plot of the unachieved moisture ratio, ln(MR) over time, utilizing the following equations^73^.

9 $$\ln \left( {MR} \right) = \ln \left( {\frac{8}{{\pi^{2} }}} \right) - \left( {\frac{{\pi^{2} \times D_{eff} \times t}}{{4L^{2} }}} \right)$$

where D_eff_ is the EMD (m^2^/s), and L is the half-thickness of n sample (m).

### Economic analysis of the developed SDET

The annualized investment cost ($C_{a}$) of the SDET was calculated using parameters in Eq. (10) as stated by^76^, Table 2 shows the calculation assumptions of economic analysis of the developed SDET.

10 $$C_{a} = C_{ac} + C_{m} - V_{a}$$

where $$C_{ac}$$ is the annualized capital cost (ACC), it was estimated according to Eq. (10); $$C_{m}$$ is the maintenance costs in $; and $$V_{a}$$ is the salvage value in $.

11 $$C_{ac} = C_{cc} \times F_{c}$$

12 $$F_{c} = \frac{{d\left( {1 + d} \right)^{n} }}{{\left( {1 + d} \right)^{n} - 1}}$$

where $$C_{cc}$$ is the total capital cost in $, $$F_{c}$$ is the capital recovery factor, $$d$$ is the interest rate in % and *n* is the operating life in years.

**Table 2:** Calculation assumptions of economic analysis of the developed SDET.

Parameter	Value
Interest rate	20%
Maintenance cost	3% of the annual capital cost
Salvage value	8% of the annual capital cost
Operating life	30 years
Inflation rate	39.7%

The drying cost per one kilogram of NTS inside the developed SDET ($C_{s}$) is calculated using Eq. (13).

13 $$C_{ds} = C_{fd} \times \frac{{M_{f} }}{{M_{d} }} + \frac{{C_{a} \times D_{d} }}{{M_{d} \times D }}$$

where $$C_{dp}$$ is the cost of dried NTS in kg, $$C_{fd}$$ is the cost of fresh NTS in $/kg, $$M_{f} \;{\text{and}}\;M_{d}$$ is the capacities of the developed SDET for fresh and dried NTS in kg, *D* is the number of operating days per year, $$D_{d}$$ is the drying days per batch.

The savings obtained from the developed SDET after (j) number of years are given by Eq. (14):

14 $$S_{j} = \frac{{\left[ {S_{pc} - C_{ds} } \right] \times M_{d} }}{D} \times D \times \left( {1 + j} \right)^{j - 1}$$

where $$S_{pc}$$ is the selling price of dried NTS in $/kg.

The payback time (Tp) for the developed SDET is calculated using Eq. (15):

15 $$Tp = \frac{{ln\left[ {1 - \frac{{C_{cc} }}{{S_{1} }}\left( {d - i} \right)} \right]}}{{\ln \left( {\frac{1 + i}{{1 + d}}} \right)}}$$

where $$i$$ is the inflation rate in %, and $$S_{1}$$ is the savings after the first year in $.

### Environmental analysis

Solar dryers are energy efficient systems that utilize readily available solar energy, which is environmentally sustainable and a clean renewable resource. The system’s energy efficiency can be evaluated by examining its overall energy consumption. Furthermore, the environmental impact of the system can be assessed by analyzing measures such as embodied energy (EE), Energy Payback Period (EPP), and greenhouse gas emissions during its life cycle. The EPP denotes the time necessary to offset the energy expended in the production of the developed SDET. The calculation is performed using Eqs. (16) and (17), as reported by Khater et al.^77^

16 $${\text{EPP}},\;{\text{years}} = { }\frac{{Input\;energy,\;{\text{kW}}}}{{{\text{Yearly}}\;{\text{energy}}\;{\text{output }}\left( {{\text{E}}_{{{\text{out}}}} } \right),\;{\text{kW}}/{\text{year}}}}$$

17 $$Daily\;energy\;outut,\;kWh = \frac{Total\;evaporative\;moisture,\; kg \times Latent\;heat\;of\;evaporation,\;J/kg}{{3.6 \times 10^{6} }}$$

The average CO_2_ emission from coal-generated energy is approximately 0.98 kg of CO_2_ per kilowatt-hour (kWh). Furthermore, transmission losses are considered, with Lt and La at 40% and 20% respectively, due to outdated equipment. The yearly CO_2_ emissions can be calculated using Eq. (18), as stated by Prakash et al.^78^:

18 $$Annual\;CO_{2} \;emissions,\;kg = \frac{EE \times 0.98}{{n }}$$

where EE is the embodied energy, kW h, n is the lifetime of the SDET, years.

The CO_2_ mitigation of the developed SDET can be estimated using Eq. (19), as mentioned by Nayak et al.^79^:

19 $$Annual\;CO_{2} \;mitigation,\;kg = EE \times n \times 1.9798$$

Each Earned Carbon Credit (ECC) represents the reduction of one metric ton of CO_2_ emissions, with the credit derived from the produced SDET calculated using Eq. (20), as illustrated by Vijayan et al.^80^:

20 $$ECC = Net\;mitigation\;of\;CO_{2} \;in\;life\;time \times Price\;per\;ton\;of\;CO_{2} \;mitigation$$

## Results and discussion

**Fig. 6:**
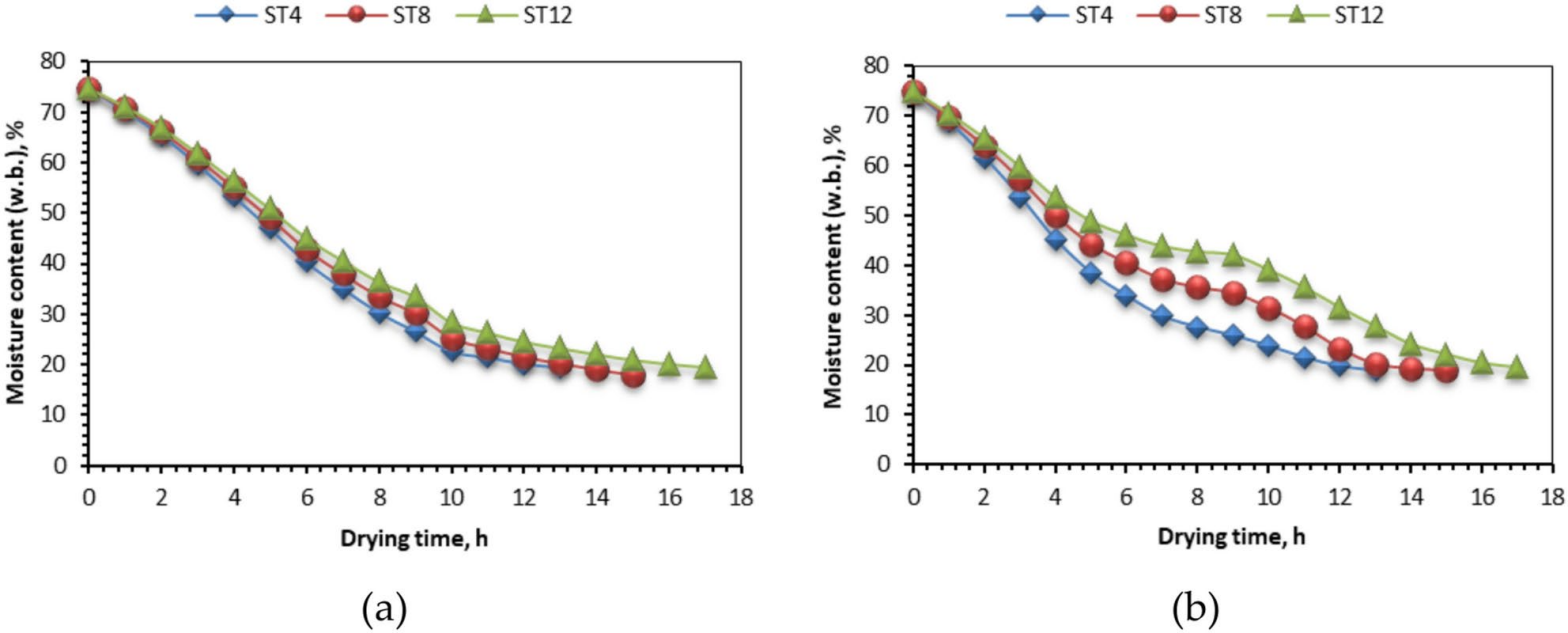
The moisture content (w.b.) of different NTS at different slice thicknesses and drying systems with respect to time. Whereas (**a**) OLPG, (**b**) SDET.

The field tests were performed in the summer of 2024, during which solar radiation values progressively increased throughout the day, peaking between 925 and 942 W/m^2^ at 1:00 p.m. The ambient temperature measurements fluctuated between 30.96 and 38.44 °C, respectively. The relative humidity of the drying air within the drying room varied from 14 to 23%. The temperature of the drying air within the drying room varied from 44 to 75 °C (Fig. 6a). The air temperature within the OLPG was sustained at 60 °C.

### Moisture content

The fluctuation of moisture content at various slice thicknesses during the experiment is illustrated visually in Fig. 6. The initial average moisture content of NTS was determined to be 74.83% on a wet basis (297.3 on a dry basis), which decreased by 18.84%, 18.8%, and 19.45% after 13, 15, and 17 h at slice thicknesses of 4 mm, 8 mm, and 12 mm, respectively, for various samples dried using SDET. The moisture content decreased by 19.8%, 17.9%, and 19.15% after 13, 15, and 17 h at slice thicknesses of 4 mm, 8 mm, and 12 mm, respectively, for various samples dried on OLPG. The study revealed that moisture content reduction during the drying process was greater at a 4 mm slice thickness, attributed to an increased volume of moisture evaporating from the NTS to the heated air within the drying chamber, which consistently diminished with prolonged drying time. The increased surface area of the NTS facilitates more efficient heat transfer from the environment to the fish, hence accelerating the evaporation process. Conversely, the moisture content consistently diminishes with drying duration due to variations in solar radiation strength and the minimal solar energy collected by the air traversing the manifold.

### Drying rate

**Fig. 7:**
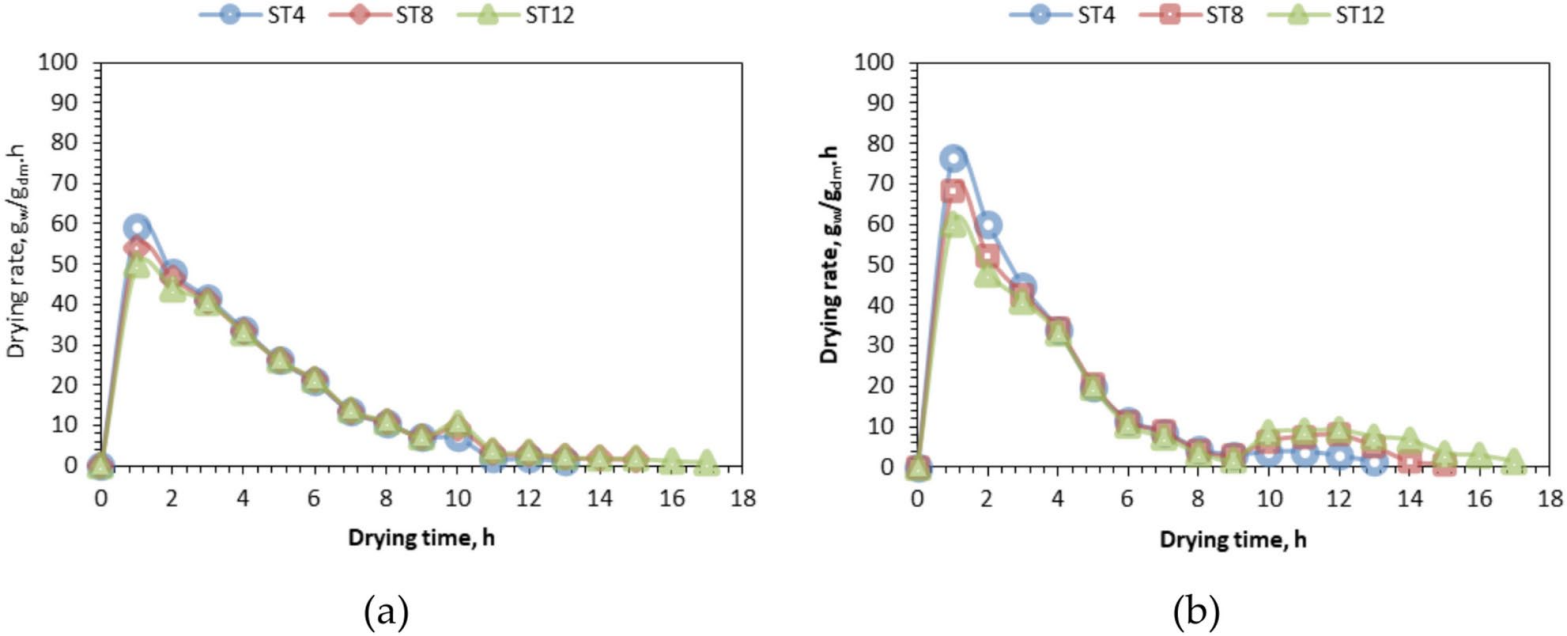
Drying rate of different NTS at different slice thicknesses and drying systems respect to time. Whereas (**a**) OLPG, (**b**), SDET, and ST refers to slice thickness.

**Fig. 8:**
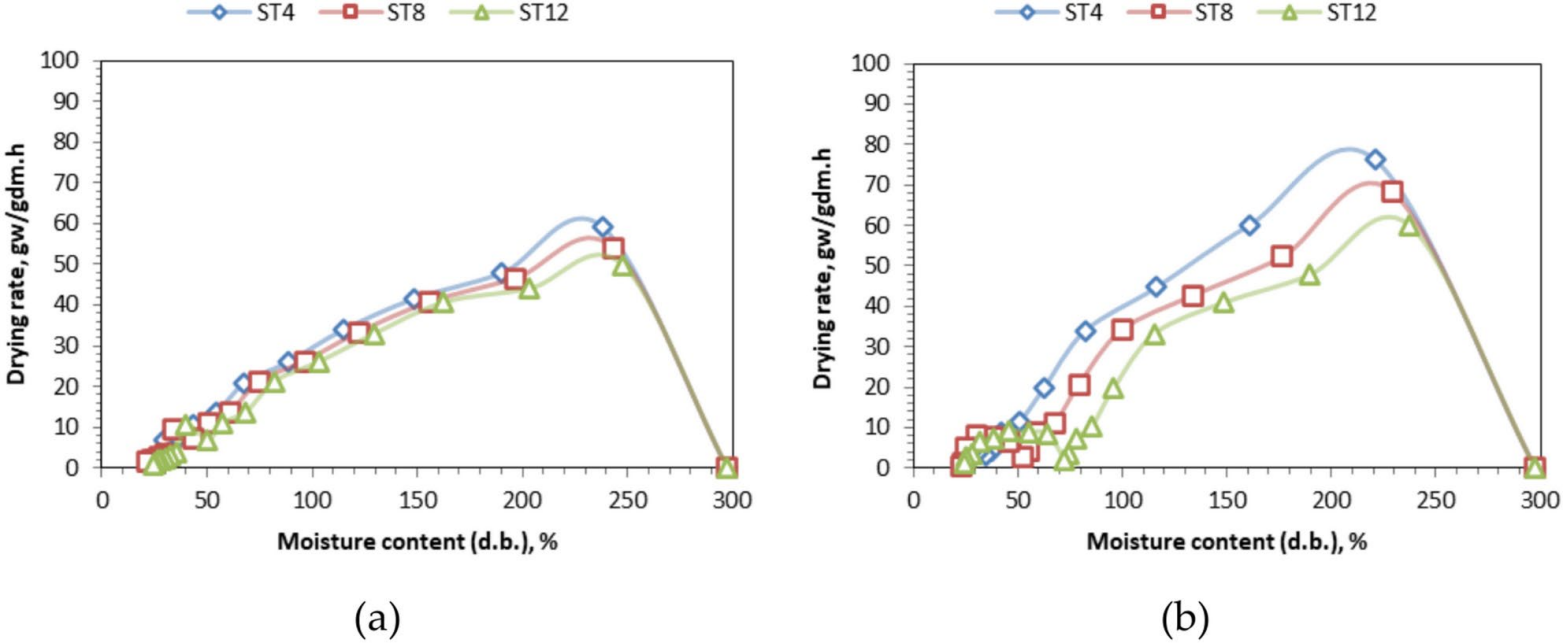
The relation between the MC and the DR at different slice thicknesses and drying systems with respect to time. Whereas (**a**) OLPG, (**b**) SDET, and ST refers to slice thickness.

Figure 7 illustrates the variance in drying rate concerning time across different slice thicknesses and drying technologies. Also, Fig. 8 shows the relation between the MC and the DR at different slice thicknesses and drying systems with respect to time. The initial drying rates were measured at 59.12 (76.33), 54.08 (68.44), and 49.88 (60.15) kg water/kg dry matter/h for slice thicknesses of 4 mm, 8 mm, and 12 mm, respectively, for OLPG (SDET). The sun intensity variation during the experiment ranged from 295 to 942 W/m^2^. The maximum drying rate recorded was 59.12 kg_water_/kg_dry matter_/h at a 4 mm slice thickness for the OLPG-dried samples, whereas the highest drying rate for the solar-dried samples was 76.33 kg_water_/kg_dry matter_/h at the same slice thickness. The results aligned with the findings reported by several papers, including those published by Elshehawy et al.^81^, where tilapia fish fillets were dehydrated at air temperatures of 40, 55, 60, and 70 °C. As the air temperature rises, the rate of moisture transfer from the inside of the product to the surface layer escalates, leading to an increased rate of moisture evaporation from the surface layer, so enhancing the drying rate. Also, Darvishi et al.^69^ indicates that the drying rate diminishes over time or with a decrease in moisture content. The product’s moisture content diminishes over time. The maximum drying rate occurred at the onset of the drying process due to the facile elimination of free moisture from the NTS, then declining consistently throughout the drying duration. The drying process was predominantly in the falling rate period across all slice thicknesses, with a brief initial phase occurring during the constant rate period. Consequently, internal mass transfer resistance is the primary factor governing the overall duration of the drying process. Additionally, the rate of moisture ratio reduction is lower at the experiment’s outset compared to the conclusion of the drying period, as indicated by Elshehawy et al.^81^.

The initial high moisture content in the tilapia fillets facilitates surface transfer and vaporization. As drying progresses, the water content within the cells diminishes markedly, complicating moisture transfer and slowing the drying process. In the final stages, the shrinkage of tilapia fillet fibers results in a substantial reduction in both moisture diffusion and drying rates, particularly at elevated temperatures. Consequently, the drying process is predominantly governed by internal diffusion.

### Moisture ratio

**Fig. 9:**
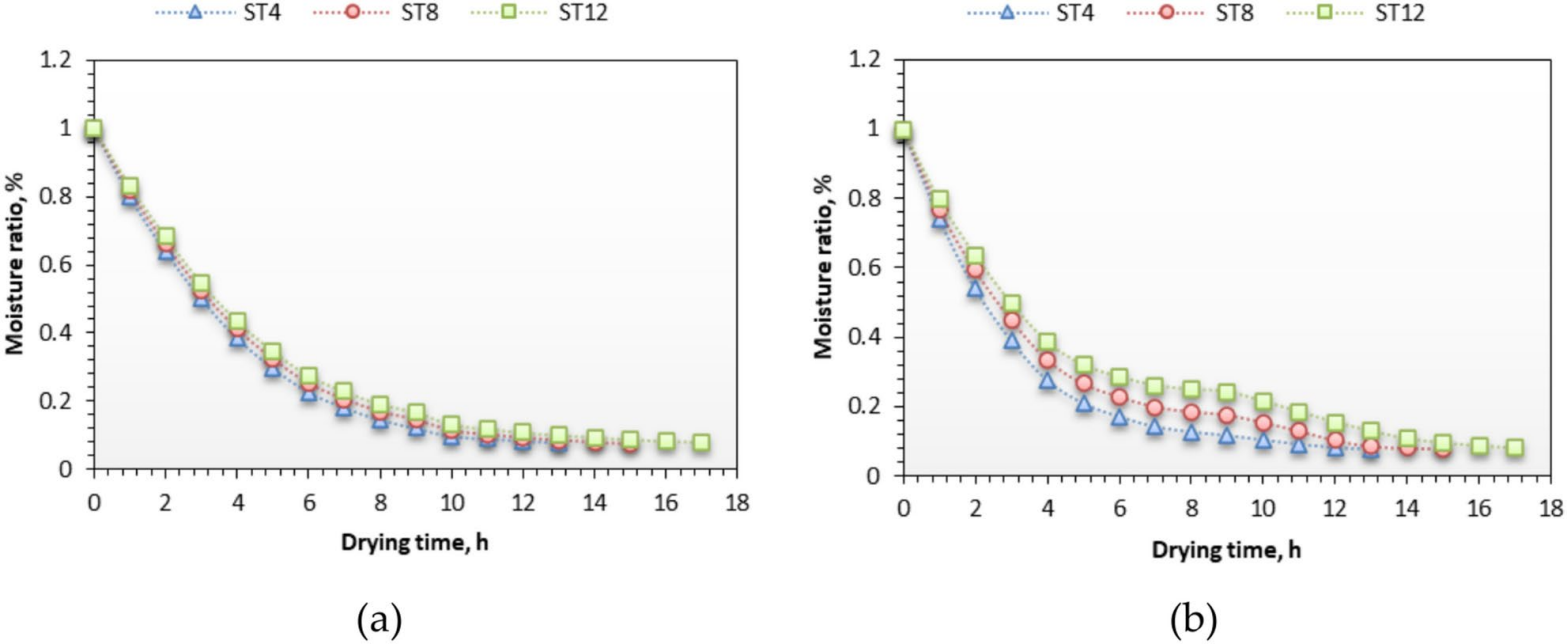
Variations of moisture ratio versus drying time of NTS at three slice thicknesses for both drying systems. Whereas (**a**) OLPG, (**b**) SDET, and ST refers to slice thickness.

Figure 9 illustrates the relationship between moisture ratio and drying time of NTS at three slice thicknesses for both drying techniques. Furthermore, Fig. 9 illustrates the fluctuations in moisture ratio across three slice thicknesses: 4 mm, 8 mm, and 12 mm. Initially, across all slice thickness levels, the moisture ratio of NTF was elevated. Increasing solar radiation from 295 to 942 W/m^2^ results in a drop in the moisture ratio. The increased free water within the samples and the elevated thermal energy absorbed by the evacuated tubes results in a reduced moisture ratio. As sun radiation diminishes, the duration required for the evaporation of moisture from the samples increases. The dehydration process of agricultural products with elevated moisture content, such as NTS, often occurs in two distinct steps. During the initial phase, moisture extraction from the NTS is elevated due to the increased moisture present on the surface of the samples. A portion of the thermal energy is utilized to evaporate moisture from the surface, while the remaining thermal energy is conveyed to the interior of the samples to elevate the temperature. Under these conditions, a capillary force exists between the internal water molecules of the samples, facilitating the internal mass capillary force, while mass transfer happens at the surface of the NTS. Additional research on the solar drying of fish slices yielded analogous findings^21,22,69,82^.

The drying duration for NTS processed by SCET ranged from 13 to 17 h to achieve the EMC. It diminished when slice thickness increased from 4 to 12 mm. The drying time for OLPG ranged from 13 to 17 h, as illustrated in Fig. 9. Conversely, employing slice thicknesses of 4 mm and 8 mm decreases the drying time by approximately 23.5% and 11.76%, respectively. The impact of employing SDET on drying time was negligible in comparison to OLPG. The samples dried in the modified system during this investigation (SCET) required a comparable duration to those dried in the industrial drying system (OLPG), which is a noteworthy accomplishment for future research considerations.

### Effective moisture diffusivity (EMD)

**Fig. 10:**
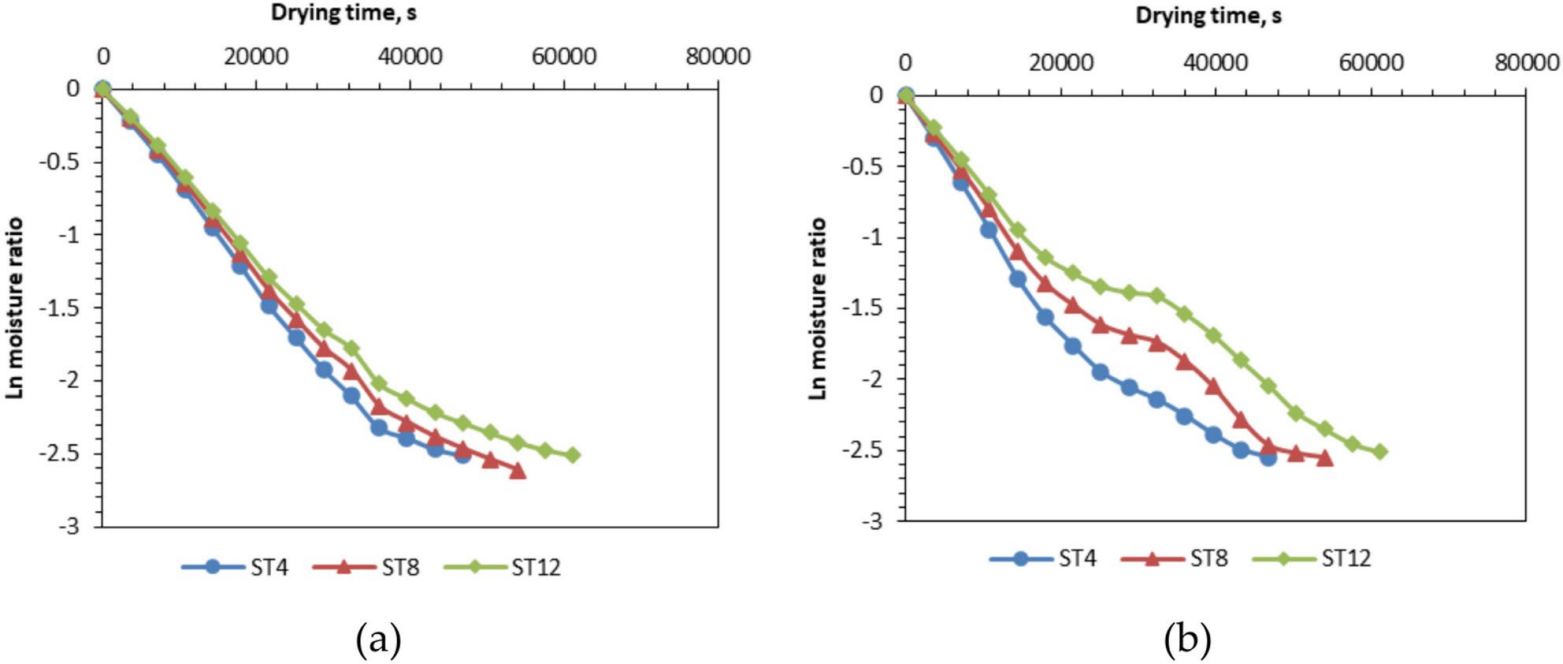
lnMR versus drying time for different drying systems and fillet thicknesses. Whereas (**a**) OLPG, (**b**) SDET, and ST refers to slice thickness.

**Fig. 11:**
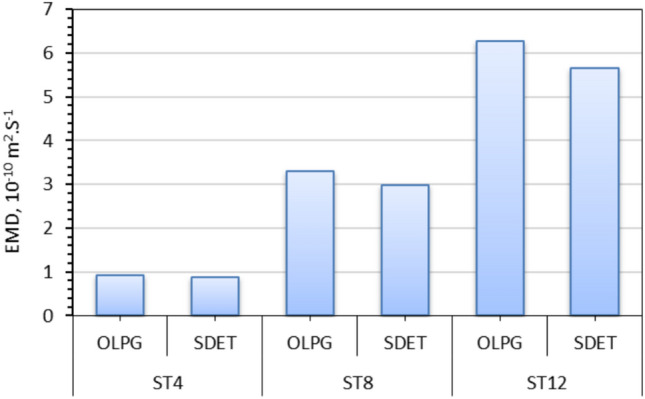
EMD of STFF for different drying systems and fillet thicknesses, where ST refers to slice thickness.

The findings indicate that internal mass transfer resistance governs the drying duration because of the existence of a declining rate drying phase. Consequently, the EMD values from the drying experiment under various conditions are computed utilizing Eq. (9) and Fig. 10 derived from Fick’s second law, as illustrated in Fig. 11 and Table 3. The effective moisture diffusivities of NTS with thicknesses of 4 mm, 8 mm, and 12 mm, at a hot air velocity of 0.50 m/s, range from 0.87 × 10^–11^ to 5.66 × 10^–11^ m^2^/s. These findings align with prior research indicating that the effective moisture diffusivities for Nile Tilapia ranged from 1.108 × 10^–11^ to 1.23 × 10^–9^ m^2^/s, as presented in Table 3. Figure 11 illustrates the influence of slice thickness and drying process on the EMD. In the identical drying method, the values of the EMDs escalate with the augmentation of the slice thickness. Numerous investigations have demonstrated that the effective diffusion coefficients of slices seemingly fluctuate with sample thickness.

**Table 3:** EMD values of some previous studies related to fish drying.

References	Dried product	EMD, m^2^/s
Guan et al.^88^	Nile Tilapia	6.55 × 10^–10^ to 1.23 × 10^–9^
Elfar et al.^81^	Nile Tilapia	3.47 × 10^–10^ to 6.86 × 10^–10^
Jain and Pathare^89^	Prawn and Chelwa fish	11.11 × 10^–11^ to 8.708 × 10^–11^
Ponwiboon and Rojanakorn^5^	Nile Tilapia	1.108 × 10^–11^ to 1.752 × 10^–11^
Boudhrioua et al.^90^	Sardine fish	7.158 × 10^–8^ to 3.408 × 10^–8^
Ghimire et al.^65^	Jaya fish	1.40 × 10^−9^ to 1.08 × 10^−8^
Darvishi et al.^69^	Sardine fish	7.158 × 10^–8^ to 3.408 × 10^–8^
Developed SDET		0.87 × 10^–11^ to 5.66 × 10^–11^

Although the effective diffusion coefficients have been found to depend on the square of the slice thickness, a theoretical explanation for this phenomenon remains unavailable as noted by Fernando et al.^83^ and Maskan et al.^84^ Aysun et al.^84^ observed that the effective diffusion coefficients rose with greater slab thicknesses. Furthermore, in a study conducted by Nguyen et al.^85^. The effective diffusion coefficient for slabs with a thickness of 2 cm has been observed to be approximately three times greater than that for slabs with a thickness of 1 cm. This discrepancy has been ascribed to the divergence between the assumption and the actual circumstances. The assumption posited that diffusion occurred unidirectionally and drying transpired solely on one side; however, in reality, diffusion occurs in both directions and drying affects all surfaces of the slabs. The assumption is only applicable for thin slabs, as the edge effect, namely moisture diffusion from the slab’s other sides, can be considered negligible in thin samples. The study highlights the significance of accounting for moisture diffusion occurring in three dimensions during the drying of thick slabs. A separate investigation elucidates that the association between thickness and effective diffusion coefficient is attributable to the contributing edge effect. The hardening effect and the potential presence of isolated air between particles in thick slices may potentially contribute to this divergence^83,85–87^. Conversely, the data provided in Fig. 11 indicated that the materials dried with OLPG demonstrated higher moisture diffusion coefficient values than those dried with the produced SDET. This gap can be ascribed to the constantly maintained temperature of 60 degrees during OLPG drying, in contrast to the changing temperature circumstances seen during SDET drying, due to the fluctuating intensity of solar radiation throughout the day, as stated by Elwakeel et al.^9^. The EMD of the dried samples on the OLPG exceeded that of the samples dried on the SDET by approximately 6.2%, 9.6%, and 9.7% at slice thicknesses of 4 mm, 8 mm, and 12 mm, respectively.

### Mathematical modeling

Eleven distinct thin-layer drying models were employed to elucidate the drying kinetics of NTS dried using both SDET and OLPG, with the results of the statistical analysis from various empirical drying models presented in Tables 4 and 5. The values of the specified mathematical models ranged from 0.983133 to 0.99988 for R^2^, 0.000030 to 0.001522 for χ^2^, 0.00314 to 0.035611 for RMSE, 0.000010 to 0.001268 for SSE, and 1.192152 to 18.77383 for E, % for dried NTS processed using SDET. The values of the specified mathematical models ranged from 0.944973 to 0.99951 for R^2^, 0.000052 to 0.004192 for χ^2^, 0.00607 to 0.059106 for RMSE, 0.000037 to 0.003494 for SSE, and 3.85738 to 27.40499 for E, % for dried NTS processed on OLPG. Additionally, Tables 4 and 5 illustrate the most suitable models for characterizing the drying behavior of NTS for OLPG and solar dryers with SDET across various slice thicknesses. The Modified Midilli (II) model was identified as the most suitable model for characterizing the drying behavior of NTS dried on OLPG across all slice thicknesses, based on the highest R^2^ and the lowest RMSE and χ^2^ values. The Modified Midilli (II) model was identified as the most suitable model for characterizing the drying behavior of NTS dried on SDET at a slice thickness of 4 mm, whilst the Modified Henderson and Pabis model was deemed most acceptable for slice thicknesses of 8 and 12 mm.

**Table 4:** Mathematical models’ constants and statistical measures results for NTS drying using OLPG under different slices thickness.

ST, mm	Model name	Constants									Statistical measures				
		k	a	b	c	d	g	h	L	n	R^2^	*χ*^2^	RMSE	SSE	E, %
4	Newton (Lewis)	0.23370									0.99739	0.00023	0.01458	0.000213	8.44842
	Page	0.23453								0.99778	0.99739	0.00025	0.01458	0.000213	8.40417
	Combined Two-Term and Page	0.23831	1.00616	0.00000				0.74762		0.99214	0.99745	0.00032	0.01442	0.000208	8.349581
	Modified Henderson and Pabis	0.16284	0.32468	0.34774	0.34133		0.27189	0.30480			0.99786	0.00031	0.01321	0.000175	7.30894
	**Modified Midilli II**	**0.21938**	**0.93700**	**0.05918**						**1.139193**	**0.99974**	**0.000030**	**0.00464**	**0.000021**	**2.04850**
	Modified Page III	1.00485				1.63262				0.62603	0.997425	0.000267	0.014491	0.000210	8.562324
	Modified Two Term III	0.23370	1.00000								0.997392	0.000248	0.014583	0.000213	8.448424
	Logistics	0.23822	34.49601	35.64725							0.99735	0.00028	0.01470	0.000216	8.81246
	Simplified Ficks Diffusion		1.00485		0.62603				1.63262		0.997425	0.000267	0.014491	0.000210	8.562324
	Approximation diffusion	0.23370	1.00	1.00							0.99739	0.00027	0.01458	0.000213	8.44842
	Parabolic model		0.95973	− 0.16808	0.00796						0.99319	0.000707	0.023566	0.000555	12.21016
8	Newton (Lewis)	0.215918									0.99647	0.00030	0.01670	0.00028	10.97187
	Page	0.22339								0.97991	0.99659	0.00031	0.01641	0.000269	10.27545
	Combined Two-Term and Page	0.23147	1.01219	0.00000				0.80013		0.96610	0.99673	0.00038	0.01608	0.000259	10.0587
	Modified Henderson and Pabis	0.21657	0.33431	0.334308	0.33431		0.21657	0.21657			0.99648	0.000445	0.01667	0.000278	11.0431
	**Modified Midilli II**	**0.20479**	**0.93590**	**0.06194**						**1.13534**	**0.99988**	**0.000013**	**0.00314**	**0.000010**	**1.19792**
	Modified Page III	1.00292				1.68070				0.61176	0.99648	0.000342	0.01667	0.000278	11.04312
	Modified Two Term III	0.21592	1.00000								0.996472	0.000319	0.01670	0.000279	10.97187
	Logistics	0.21731	20,529.02	20,657.31							0.99647	0.00034	0.01671	0.000279	11.12284
	Simplified Ficks Diffusion		1.00292		0.61176				1.68070		0.996483	0.000342	0.016672	0.000278	11.04312
	Approximation diffusion	0.21592	1.00	1.00							0.99647	0.00034	0.01670	0.000279	10.97187
	Parabolic model		0.94759	− 0.14911	0.00628						0.98841	0.001127	0.030263	0.000916	16.90424
12	Newton (Lewis)	0.198211									0.99270	0.00058	0.02343	0.00055	15.23128
	Page	0.21889								0.94406	0.99372	0.00053	0.02174	0.000473	12.88814
	Combined Two-Term and Page	0.23110	1.01900	0.00000				0.74436		0.92417	0.99403	0.00062	0.02119	0.000449	12.5566
	Modified Henderson and Pabis	0.19753	0.33225	0.33225	0.33225		0.19753	0.19753			0.99272	0.00082	0.02341	0.000548	15.14199
	**Modified Midilli II**	**0.19155**	**0.92272**	**0.075855**						**1.14790**	**0.99985**	**0.000014**	**0.003354**	**0.000011**	**1.192152**
	Modified Page III	0.99676				1.72440				0.58738	0.992716	0.000658	0.023409	0.000548	15.14199
	Modified Two Term III	0.19821	1.00000								0.99270	0.00062	0.02343	0.000549	15.23128
	Logistics	0.19812	419.87	419.74							0.99269	0.00066	0.02345	0.000550	15.22594
	Simplified Ficks Diffusion		0.99676		0.58738				1.72440		0.992716	0.000658	0.023409	0.000548	15.14199
	Approximation diffusion	0.19821	1.00	1.00							0.99270	0.00066	0.02343	0.000549	15.23128
	Parabolic model		0.93653	− 0.13367	0.00511						0.983133	0.001522	0.035611	0.001268	18.77383

Significant values are in bold.

**Table 5:** Mathematical models’ constants and statistical measures results for NTS drying using SDET under different slices thickness.

ST, mm	Model name	Constants									Statistical measures				
		k	a	b	c	d	g	h	L	n	R^2^	*χ*^2^	RMSE	SSE	E,%
4	Newton (Lewis)	0.28902									0.98394	0.00129	0.03462	0.00120	24.35329
	Page	0.35632								0.85171	0.99091	0.00079	0.02602	0.000677	15.71114
	Combined Two-Term and Page	0.24160	0.65942	0.42720				0.31885		0.94185	0.99248	0.00087	0.02366	0.000560	14.39044
	Modified Henderson and Pabis	0.01322	0.08186	0.413929	0.51578		0.39809	0.34568			0.99857	0.000186	0.01032	0.000106	3.87276
	**Modified Midilli II**	**0.32937**	**0.91660**	**0.084044**						**1.099653**	**0.99951**	**0.000052**	**0.00607**	**0.000037**	**3.85738**
	Modified Page III	0.97700				1.54033				0.66867	0.984645	0.001459	0.033852	0.001146	23.46553
	Modified Two Term III	0.28902	1.00000								0.98394	0.00140	0.034616	0.001198	24.35329
	Logistics	0.28217	3717.21	3639.4							0.98463	0.00146	0.03386	0.001147	23.49855
	Simplified Ficks Diffusion		0.97700		0.66867				1.54033		0.984645	0.001459	0.033852	0.001146	23.46553
	Approximation diffusion	0.28902	1.00	1.00							0.98394	0.001525	0.034616	0.001198	24.35329
	Parabolic model		0.90410	− 0.17600	0.00908						0.963216	0.003487	0.052339	0.002739	27.40499
8	Newton (Lewis)	0.23421									0.97600	0.001761	0.040631	0.001651	24.26645
	Page	0.32160								0.80509	0.99148	0.00067	0.02419	0.000585	10.35877
	Combined Two-Term and Page	0.30978	0.96318	0.052747				0.411063		0.814456	0.99182	0.00082	0.02370	0.000562	10.45093
	**Modified Henderson and Pabis**	**0.07815**	**0.25389**	**0.343745**	**0.41363**		**0.38103**	**0.36647**			**0.99712**	**0.00032**	**0.014067**	**0.000198**	**6.45389**
	Modified Midilli II	0.31071	0.92479	0.080789						0.977067	0.99626	0.000343	0.016032	0.000257	8.714082
	Modified Page III	0.95473				1.65769				0.61110	0.97888	0.001789	0.038122	0.001453	21.76363
	Modified Two Term III	0.23421	1.00000								0.97600	0.00189	0.04063	0.001651	24.26645
	Logistics	0.22572	7085.33	6850.41							0.97867	0.00181	0.03831	0.001468	22.33869
	Simplified Ficks Diffusion		0.95473		0.61110				1.65769		0.978881	0.001789	0.038122	0.001453	21.76363
	Approximation diffusion	0.23421	1.00	1.00							0.97600	0.00203	0.04063	0.001651	24.26645
	Parabolic model		0.88758	− 0.14115	0.00611						0.953644	0.003919	0.056427	0.003184	24.45569
12	Newton (Lewis)	0.18896									0.96216	0.00255	0.04903	0.00240	23.00661
	Page	0.29054								0.76610	0.98961	0.00074	0.02569	0.000660	7.00268
	Combined Two-Term and Page	0.28550	0.97761	0.039659				0.55590		0.76823	0.98993	0.000885	0.02528	0.000639	7.02288
	**Modified Henderson and Pabis**	**0.10490**	**0.29684**	**0.220726**	**0.49575**		**0.10343**	**0.46559**			**0.99411**	**0.00056**	**0.01934**	**0.000374**	**7.48202**
	Modified Midilli II	0.29644	0.94898	0.062334						0.84984	0.991257	0.000714	0.023561	0.000555	9.637797
	Modified Page III	0.92982				1.79757				0.56178	0.969778	0.002306	0.043833	0.001921	18.76276
	Modified Two Term III	0.18896	1.00000								0.96216	0.00270	0.04903	0.002404	23.00661
	Logistics	0.17291	4303.42	3980.14							0.96973	0.00231	0.04387	0.001925	18.51381
	Simplified Ficks Diffusion		0.92982		0.56178				1.79757		0.969778	0.002306	0.043833	0.001921	18.76276
	Approximation diffusion	0.18896	1.00	1.00							0.962157	0.002885	0.049034	0.002404	23.00661
	Parabolic model		0.87417	− 0.11395	0.00417						0.944973	0.004192	0.059106	0.003494	21.85639

Significant values are in bold.

The outcomes obtained from the Modified Midilli (II) and Modified Henderson and Pabis models closely align with the experimental values. The prediction via the thin layer drying model validated that the MR values aligned along a straight line, hence confirming the suitability of the models for elucidating the drying behavior of NTS.

### Environmental analysis of the SDET

Embodied energy (EE) is the total energy required to generate goods or services, encompassing the energy needed for the extraction and processing of natural resources, as well as production, transportation, and product delivery^91^. Table 6 presents the energy efficiency of the different materials employed in the production of the designed SDET.

**Table 6:** EE calculation data for manufacturing of the developed SDET.

S. no.	Component	Material	EE	Weight	Total EE, kWh	References
1	Drying room	Wood	2.0 kWh/kg	15 kg	30.0	^92^
2	Hinges	Metal	8.89 kWh/kg	0.1 kg	0.889	^93^
3	Handel	Metal	8.89 kWh/kg	0.1 kg	0.889	^93^
4	Drying trays	Metal	8.89 kWh/kg	2.0 kg	17.72	^93^
5	Evacuated tubes	–	1138.52 kWh/tube	4 tubes	4552	^94^
6	Air circulation fan					
	(i) Copper wire	Copper	19.61 kWh/kg	0.2 kg	3.922	^93^
	(ii) Casings, fan, shaft etc	Steel	8.89 kWh/kg	0.2 kg	1.778	^93^
Total EE, kWh					4607.198	

Table 6 indicates that the total energy expenditure necessary for the established SDET is 4607.198 kW/h. The evacuated tubes constituted the primary value of the EE, comprising 99% of the total EE (4552 kW h). The created SFCSD eliminates approximately 1 kg of moisture for 10 h daily. The dryer operates for 10 h daily, from 07:00 to 17:00. Nonetheless, the operational period may vary according to the season. The typical evaporation capacity of a dryer is estimated to be 1 kg per day. The latent heat of vaporization of water is ascribed to a value of 2257 kJ/kg. The energy payback period for the developed SDET is calculated to be 6.46 years. The present duration is markedly shorter than the lifespan of the conventional solar dryer (30 years). The annual CO_2_ emissions, CO_2_ mitigation, and carbon credits earned (CCE) were computed over a period of 5–30 years, with the results displayed in Table 7. The CCE is $ 72.50 per metric ton of CO_2_^95^. Furthermore, Table 7 displayed the CCE achieved by the created SDET throughout various durations of 5, 10, 15, 20, and 30 years. The results indicate that the designed SDET can mitigate approximately 273.6 tons of CO_2_ over its lifespan, resulting in a CCE of roughly 19,838.89 $.

**Table 7:** CO_2_ mitigation (Tons) for the developed SDET.

Item	5 years	10 years	15 years	20 years	25 years	30 years
Carbon footprint emission (Kg/year)	903.01	451.51	301.00	225.75	180.60	150.50
Carbon footprint mitigation (Tons)	45.6	91.2	136.8	182.4	228.0	273.6
CCE, $	3306.48	6612.96	9919.45	13,225.93	16,532.41	19,838.89

### Economic analysis of the SDET

The performance analysis of SDET entails a quantitative assessment of economic processes that can aid policymakers, investors, and food processors in making educated decisions regarding NTS drying systems. In the economic analysis of the developed SDET, numerous parameters were considered, all of which are enumerated in Table 8 based on the condition of the Egyptian economy and the projected costs related to the SDET components. Table 8 demonstrates that the capital cost of the DSD is about 200 $, a much lower in comparison to alternative drying systems, with an anticipated lifespan of 30 years. The yearly capital cost was 40.17 $, whereas the annual investment cost was 38.16 $. The data in the table indicates that the designed SDET can produce 450 kg of dry NTS annually. This might yield significant cost reductions, totaling 608.4 $ annually. Moreover, the study indicated that the payback period of the created SDET was around 0.413 years, or under five months, so illustrating its cost-effectiveness.

**Table 8:** Economic analysis of the developed SDET for drying NTS.

Parameters	Unit
Capital cost	200 $
Annual capital cost	40.17 $
Annualized investment cost	38.16 $
Mass of NTS per batch	5.0 kg
Quantity of dried NTS annually	1800 kg
Drying cost per kg of NTS	0.0212 $
Cost of 1 kg fresh NTS	0.412 $
Cost of fresh NTS per kg of dried product	1.648 $
Selling price per kg of NTS	3.0 $
Saving after 1 year	608.4 $
Payback time	0.413 year

## Conclusion and future considerations

In the present study a low-carbon footprint solar dryer with evacuated tube (SDET) was used for drying Nile Tilapia fish fillets compared with an industrial method of oven liquefied petroleum gas (OLPG). In summer 2024, the two drying systems were compared at 0.5 m/s and 4, 8, and 12 mm slice thicknesses. The results showed that both systems dried in 13–15 h. As slice thickness increased from 4 to 12 mm, OLPG and SDET drying rates decreased from 59.12 to 49.88 and 76.33 to 60.15 kg_water_/kg_dry matter_/h, respectively. The effective moisture diffusivity (EMD) ranging from 0.87 × 10^–11^ to 5.66 × 10^–11^ m^2^/s. The maximum EMD of dried samples on the OLPG was 9.7% higher than that of SDET samples at slice thicknesses of 12 mm. The Modified Midilli (II) and Henderson and Pabis models match SDET experimental values. Conversely, the SDET environmental assessment showed 4607.198 kW.h of energy expenditure. The designed SDET has a 6.46-year energy payback. The SDET may reduce 273.6 tons of CO_2_ over its lifetime, saving 19,838.89 $. In addition, the SDET’s economic assessment estimated a $200 total capital cost and a $40.17 annual capital cost. This might save 608.4 $ per year, reducing the payback period to 0.413 years. Evacuated tubes made a low-carbon footprint solar dryer for drying Nile Tilapia slices more efficient, reducing capital and annual costs. Considerations for the Future: The next phase of development will integrate machine learning and artificial intelligence into the solar dryer system to accurately forecast processed product drying characteristics.

## Data Availability

“All data are provided within the article”.

## Abbreviations

NTS: Nile Tilapia slice
SDET: Solar dryer with evacuated tubes
OLPG: Oven liquid petroleum gas
MC: Moisture content
w.b.: Wet base
PCM: Phase change material
SC: Solar collector
TE: Thermal efficiency
ETSC: Evacuated tube solar collector
MR: Moisture ratio
DR: Drying rate
ACC: Annualized capital cost
EE: Embodied energy
EPP: Energy payback period
ECC: Earned carbon credit

## List of symbols

*D*: Number of operating days per year
$$D_{d}$$: Drying days per batch
$$SP_{c}$$: Selling price of dried Nile Tilapia slice in $/kg
$$i$$: Inflation rate in %
$$S_{1}$$: Savings after the first year in $
$$C_{dp}$$: Cost of dried nile Tilapia slice in kg
$$C_{fd}$$: Cost of fresh nile Tilapia slice in $/kg
M_t_: Moisture content at any time of drying in (kg_water_/kg_dry matter_)
M_0_: Initial moisture content in (kg_water_/kg_dry matter_)
M_e_: Equilibrium moisture content in (kg_water_/kg_dry matter_)
M_(t+dt)_: Moisture content at (t + dt)
t: Drying time in h
R^2^: Coefficient of determination
*χ*^2^: Chi-square
RMSE: Root mean square error
SSE: Sum square error
E: Average percentage error in %
MR_exp,i_: Experimental moisture ratio
MR_pre,i_: Predicted moisture ratio
N: Number of observations
n: Number of constants
D_eff_: Effective moisture diffusivity in m^2^/s
L: Half-thickness in m
$$C_{a}$$: Annualized investment cost
$$C_{m}$$: Maintenance costs in $
$$V_{a}$$: Salvage value in $
$$C_{cc}$$: Total capital cost in $
$$F_{c}$$: Capital recovery factor
$$d$$: Interest rate in %
*n*: Operating life in years
$$C_{s}$$: Drying cost per one kilogram
$$M_{f} \;{\text{and}}\;M_{d}$$: Capacities of the developed solar dryer with evacuated tubes for fresh and dried nile Tilapia slice in kg

## References

[1] Jim, F., Garamumhango, P. & Musara, C. Comparative analysis of nutritional value of *Oreochromis niloticus* (Linnaeus), Nile tilapia, meat from three different ecosystems. *J. Food Qual.* **2017**, 6714347 (2017).

[2] El-Sayed, A. M. & Fitzsimmons, K. From Africa to the world: The journey of Nile tilapia. *Rev. Aquac.* **15**, 6–21 (2023).

[3] Natarajan, S. K., Muthuvairavan, G., Suraparaju, S. K., Elangovan, E. & Samykano, M. Innovative insights into solar drying of Kola fish: Mechanisms, modeling, and optimization. *Appl. Sol. Energy* **59**, 887–902 (2023).

[4] Duan, Z., Jiang, L., Wang, J., Yu, X. & Wang, T. Drying and quality characteristics of tilapia fish fillets dried with hot air-microwave heating. *Food Bioprod. Process.* **89**, 472–476 (2011).

[5] Ponwiboon, N. & Rojanakorn, T. Desorption isotherms and drying characteristics of Nile tilapia fish sheet. *Int. Food Res. J.* **24**, 1292–1300 (2017).

[6] Alemu, L. A., Melese, A. Y. & Gulelat, D. H. Effect of endogenous factors on proximate composition of nile tilapia (*Oreochromis niloticus* L.) fillet from lake zeway. *Am. J. Res. Commun.* **1**, 405–410 (2013).

[7] Iranmanesh, M., Samimi, H., Saleh, M. & Jahromi, B. CFD modeling and evaluation the performance of a solar cabinet dryer equipped with evacuated tube solar collector and thermal storage system. *Renew. Energy* **145**, 1192–1213 (2020).

[8] Eissa, A. S., Gameh, M. A., Mostafa, M. B. & Elwakeel, A. E. Some engineering factors affecting utilization of solar energy in drying tomato fruits introduction. *Aswan Univ. J. Environ. Stud.* **5**, 52–68 (2024).

[9] Elwakeel, A. E., Gameh, M. A., Eissa, A. S. & Mostafa, M. B. Recent advances in solar drying technology for tomato fruits: A comprehensive review. *Int. J. Appl. Energy Syst.* **6**, 37–44 (2024).

[10] Muthuvairavan, G., Manikandan, S., Elangovan, E. & Natarajan, S. K. Assessment of solar dryer performance for drying different food materials: A comprehensive review (2023).

[11] Suraparaju, S. K. et al. Assessing thermal and economic performance of solar dryers in sustainable strategies for bottle gourd and tomato preservation. *Sci. Rep.* **14**, 27755 (2024). 39532916 10.1038/s41598-024-78147-2 PMC11557917

[12] Elavarasan, E., Natarajan, S. K., Bhanu, A. S., Anandu, A. & Senin, M. H. Experimental investigation of drying cucumber in a double slope solar dryer under natural convection and open sun drying. In *Innovations in Energy, Power and Thermal Engineering: Select Proceedings of ICITFES 2020* 41–52 (Springer, 2022).

[13] Motahayyer, M., Arabhosseini, A., Samimi-Akhijahani, H. & Khashechi, M. Application of computational fluid dynamics in optimization design of absorber plate of solar dryer. *Iran. J. Biosyst. Eng.* **49**, 285–294 (2018).

[14] Elavarasan, E., Kumar, Y., Mouresh, R. & Natarajan, S. K. Experimental investigation of drying tomato in a double slope solar dryer under natural convection. In *Advances in Mechanical and Materials Technology: Select Proceedings of EMSME 2020* 179–190 (Springer, 2022).

[15] Arabhosseini, A., Samimi-Akhijahani, H. & Motahayyer, M. Increasing the energy and exergy efficiencies of a collector using porous and recycling system. *Renew. Energy* **132**, 308–325 (2019).

[16] Kumar, M., Sahdev, R. K., Tawfik, M. A. & Elboughdiri, N. Natural convective greenhouse vermicelli drying: Thermo-environ-econo-kinetic analyses. *Sustain. Energy Technol. Assess.* **55**, 103002 (2023).

[17] Wang, P. et al. High temperature collecting performance of a new all-glass evacuated tubular solar air heater with U-shaped tube heat exchanger. *Energy Convers. Manag.* **77**, 315–323 (2014).

[18] Aggarwal, S., Kumar, R., Lee, D., Kumar, S. & Singh, T. A comprehensive review of techniques for increasing the efficiency of evacuated tube solar collectors. *Heliyon* **9**, e15185 (2023). 37089311 10.1016/j.heliyon.2023.e15185 PMC10114210

[19] Elangovan, E. & Natarajan, S. K. Experimental research of drying characteristic of red banana in a single slope direct solar dryer based on natural and forced convection. *Food Technol. Biotechnol.* **59**, 137–146 (2021). 34316275 10.17113/ftb.59.02.21.6876 PMC8284107

[20] Belton, B. et al. Dried fish at the intersection of food science, economy, and culture: A global survey. *Fish Fish.* **23**, 941–962 (2022).

[21] Elwakeel, A. E. et al. Design and implementation of a PV-integrated solar dryer based on internet of things and date fruit quality monitoring and control. *Int. J. Energy Res.* **2023**, 7425045 (2023).

[22] Kituu, G. M. Influence of brining on the drying parameters of tilapia (*Oreochromis niloticus*) in a glass-covered solar tunnel dryer. *Agric. Eng. Int. CIGR J.* **11**, 88–98 (2009).

[23] Elavarasan, E., Bhanu, A. S., Kumar, Y., Mouresh, R. & Natarajan, S. K. Energy and exergy analysis of poovan banana under single slope forced and natural convection solar drying. In *International Conference on Energy, Materials Sciences & Mechanical Engineering* 321–331 (Springer, 2020).

[24] Metwally, K. A. et al. The mathematical modeling, diffusivity, energy, and enviro-economic analysis (MD3E) of an automatic solar dryer for drying date fruits. *Sustainability* **16**, 3506 (2024).

[25] Natarajan, S. K. & Elavarasan, E. A review on computational fluid dynamics analysis on greenhouse dryer. In *IOP Conference Series: Earth and Environmental Science* vol. 312 012033 (IOP Publishing, 2019).

[26] Elangovan, E. & Natarajan, S. K. Convective and evaporative heat transfer coefficients during drying of ivy gourd under natural and forced convection solar dryer. *Environ. Sci. Pollut. Res.* **30**, 10469–10483 (2023). 10.1007/s11356-022-22865-5 36074290

[27] Natarajan, S. K. & Elavarasan, E. Experimental investigation of drying potato for Karaikal climatic condition. In *IOP Conference Series: Earth and Environmental Science* vol. 312 012021 (IOP Publishing, 2019).

[28] Research Nester. Solar dryer market size & share, statistical report 2025–2037. https://www.researchnester.com/reports/solar-dryer-market/5439#:~:text=Solar%20Dryer%20Market%20size%20was,than%205.8%25%20CAGR%20during%20the (2025).

[29] Zeeshan, M. et al. Novel design and performance evaluation of an indirectly forced convection desiccant integrated solar dryer for drying tomatoes in Pakistan. *Heliyon* **10**, e29284 (2024). 38655325 10.1016/j.heliyon.2024.e29284 PMC11036014

[30] Elangovan, E. & Natarajan, S. K. Study of activation energy and moisture diffusivity of various dipping solutions of ivy gourd using solar dryer. *Environ. Sci. Pollut. Res.* **30**, 996–1010 (2023). 10.1007/s11356-022-22248-w 35907071

[31] Fudholi, A. & Sopian, K. A review of solar air flat plate collector for drying application. *Renew. Sustain. Energy Rev.* **102**, 333–345 (2019).

[32] El Khadraoui, A., Bouadila, S., Kooli, S., Farhat, A. & Guizani, A. Thermal behavior of indirect solar dryer: Nocturnal usage of solar air collector with PCM. *J. Clean Prod.* **148**, 37–48 (2017).

[33] Das, S. K. & Kumar, Y. Design and performance of a solar dryer with vertical collector chimney suitable for rural application. *Energy Convers. Manag.* **29**, 129–135 (1989).

[34] Abhay, L., Chandramohan, V. P. & Raju, V. R. K. Numerical analysis on solar air collector provided with artificial square shaped roughness for indirect type solar dryer. *J. Clean. Prod.* **190**, 353–367 (2018).

[35] Ebrahimi, H., Akhijahani, H. S. & Salami, P. Improving the thermal efficiency of a solar dryer using phase change materials at different position in the collector. *Sol. Energy* **220**, 535–551 (2021).

[36] Nasrat, L. S., Badawy, M. E., Ourapi, M. A. & Elwakeel, A. E. Some engineering factors affecting the performance of an automatic sugarcane seed cutting machine 1-introduction. *Aswan Univ. J. Environ. Stud.* **5**, 87–100 (2024).

[37] El-Sebaii, A. A., Aboul-Enein, S., Ramadan, M. R. I., Shalaby, S. M. & Moharram, B. M. Investigation of thermal performance of-double pass-flat and v-corrugated plate solar air heaters. *Energy* **36**, 1076–1086 (2011).

[38] Natarajan, S. K., Suraparaju, S. K., Muthuvairavan, G., Elangovan, E. & Samykano, M. Experimental analysis and development of novel drying kinetics model for drying grapes in a double slope solar dryer. *Renew. Energy* **236**, 121508 (2024).

[39] Elangovan, E. & Natarajan, S. K. Effect of pretreatments on drying of red dacca in a single slope solar dryer. *J. Food Process Eng.* **44**, e13823 (2021).

[40] Elangovan, E. & Natarajan, S. K. Physicochemical characteristics of dried ivy gourd in a single slope solar dryer. *Environ. Prog. Sustain. Energy* **41**, e13812 (2022).

[41] Bhowmik, H. & Amin, R. Efficiency improvement of flat plate solar collector using reflector. *Energy Rep.* **3**, 119–123 (2017).

[42] Belusko, M., Saman, W. & Bruno, F. Performance of jet impingement in unglazed air collectors. *Sol. Energy* **82**, 389–398 (2008).

[43] Karim, M. A. & Hawlader, M. Development of solar air collectors for drying applications. *Energy Convers. Manag.* **45**, 329–344 (2004).

[44] Kumar, M. et al. Experimental forced convection greenhouse and indirect cabinet drying of date fruits: a comparative study. *J. Therm. Anal. Calorim.* **148**, 5437–5454 (2023).

[45] Tang, R., Li, Z., Zhong, H. & Lan, Q. Assessment of uncertainty in mean heat loss coefficient of all glass evacuated solar collector tube testing. *Energy Convers. Manag.* **47**, 60–67 (2006).

[46] ElGamal, R., Kishk, S., Al-Rejaie, S. & ElMasry, G. Incorporation of a solar tracking system for enhancing the performance of solar air heaters in drying apple slices. *Renew. Energy* **167**, 676–684 (2021).

[47] Zheng, W., Yang, L., Zhang, H., You, S. & Zhu, C. Numerical and experimental investigation on a new type of compound parabolic concentrator solar collector. *Energy Convers. Manag.* **129**, 11–22 (2016).

[48] Chamsa-ard, W., Sukchai, S., Sonsaree, S. & Sirisamphanwong, C. Thermal performance testing of heat pipe evacuated tube with compound parabolic concentrating solar collector by ISO 9806–1. *Energy Procedia* **56**, 237–246 (2014).

[49] Rittidech, S., Donmaung, A. & Kumsombut, K. Experimental study of the performance of a circular tube solar collector with closed-loop oscillating heat-pipe with check valve (CLOHP/CV). *Renew. Energy* **34**, 2234–2238 (2009).

[50] Wei, L., Yuan, D., Tang, D. & Wu, B. A study on a flat-plate type of solar heat collector with an integrated heat pipe. *Sol. Energy* **97**, 19–25 (2013).

[51] Verma, S. K., Sharma, K., Gupta, N. K., Soni, P. & Upadhyay, N. Performance comparison of innovative spiral shaped solar collector design with conventional flat plate solar collector. *Energy* **194**, 116853 (2020).

[52] Ramachandran, S., Nene, A. A. & Suyambazhahan, S. Integrated system of flat plate collector and Scheffler solar concentrator for enhancing thermal efficiency and steam generation rate. *Int. J. Ambient Energy* **43**, 3154–3163 (2022).

[53] Das, M. & Akpinar, E. K. Determination of thermal and drying performances of the solar air dryer with solar tracking system: Apple drying test. *Case Stud. Therm. Eng.* **21**, 100731 (2020).

[54] Ismail, M. A. *et al.* Improving the performance of solar panels by the used of dual axis solar tracking system with mirror reflection. In *Journal of Physics: Conference Series* vol. 1432 12060 (IOP Publishing, 2020).

[55] Puente-Díaz, L., Ah-Hen, K., Vega-Gálvez, A., Lemus-Mondaca, R. & Di Scala, K. Combined infrared-convective drying of murta (*Ugni molinae* Turcz) berries: Kinetic modeling and quality assessment. *Dry. Technol.* **31**, 329–338 (2013).

[56] Elavarasan, E., Kumar, Y., Mouresh, R. & Natarajan, S. K. Study of drying kinetics of tomato in a solar dryer. In *Current Advances in Mechanical Engineering: Select Proceedings of ICRAMERD 2020* 349–358 (Springer, 2021).

[57] Kushwah, A., Kumar, A., Gaur, M. K. & Pal, A. Heat and mass transfer, quality, performance analysis, and modeling of thin layer drying kinetics of banana slices. *J. Sol. Energy Eng.* **145**, 051010 (2023).

[58] Kushwah, A., Kumar, A. & Gaur, M. K. Optimization of drying parameters for hybrid indirect solar dryer for banana slices using response surface methodology. *Process Saf. Environ. Prot.* **170**, 176–187 (2023).

[59] Mahesh, S. & Anil, K. Performance assessment and modeling techniques for domestic solar dryers. *Food Eng. Rev.*10.1007/s12393-023-09335-5 (2023). 10.1007/s12393-023-09335-5 PMC9887261 40479461

[60] Zhou, Y.-H. et al. Microwave-vacuum-assisted drying of pretreated cranberries: Drying kinetics, bioactive compounds and antioxidant activity. *LWT* **146**, 111464 (2021).

[61] An, N. et al. Effect of different drying techniques on drying kinetics, nutritional components, antioxidant capacity, physical properties and microstructure of edamame. *Food Chem.* **373**, 131412 (2022). 34731799 10.1016/j.foodchem.2021.131412

[62] Ortiz, J. et al. Influence of air-drying temperature on drying kinetics, colour, firmness and biochemical characteristics of Atlantic salmon (*Salmo salar* L.) fillets. *Food Chem.* **139**, 162–169 (2013). 23561093 10.1016/j.foodchem.2013.01.037

[63] Cao, F., Zhang, R., Tang, J., Li, F. & Jiao, Y. Radio frequency combined hot air (RF-HA) drying of tilapia (*Oreochromis niloticus* L.) fillets: Drying kinetics and quality analysis. *Innov. Food Sci. Emerging Technol.* **74**, 102791 (2021).

[64] Liu, J., Zhao, Y., Shi, Q., Wu, X. & Fang, Z. Water distribution, physicochemical and microstructural properties of scallop adductors as affected by different drying methods. *J. Food Compos. Anal.* **115**, 104966 (2023).

[65] Ghimire, A., Basnet, S., Poudel, R. & Ghimire, A. Mathematical modeling of thin layer microwave drying of Jaya fish (*Aspidoparia jaya*). *Food Sci. Technol. Int.* **27**, 508–516 (2021). 33143468 10.1177/1082013220969353

[66] Wu, Y. Y. et al. Mathematical modeling of drying kinetics of salted *Otolithes ruber* at the different temperature. *Adv. Mater. Res.* **781**, 1347–1352 (2013).

[67] AOAC. *Official Method of Analysis*. (Association of Officiating Analytical Chemists, Washington, 2005).

[68] Basiouny, M. A. Thin layer drying of nile tilapia fingerlings using mechanical dryer. *Misr J. Agric. Eng.* **31**, 599–618 (2014).

[69] Darvishi, H., Azadbakht, M., Rezaeiasl, A. & Farhang, A. Drying characteristics of sardine fish dried with microwave heating. *J. Saudi Soc. Agric. Sci.* **12**, 121–127 (2013).

[70] Al-Harahsheh, M., Ala’a, H. & Magee, T. R. A. Microwave drying kinetics of tomato pomace: Effect of osmotic dehydration. *Chem. Eng. Process. Process Intensif.* **48**, 524–531 (2009).

[71] Doymaz, İ. Drying characteristics and kinetics of okra. *J. Food Eng.* **69**, 275–279 (2005).

[72] Inyang, U. E., Oboh, I. O. & Etuk, B. R. Kinetic models for drying techniques: Food materials. *Adv. Chem. Eng. Sci.*10.4236/aces.2018.82003 (2018).

[73] Crank, J. *The Mathematics of Diffusion* (Oxford University Press, 1975).

[74] Rabha, D. K., Muthukumar, P. & Somayaji, C. Experimental investigation of thin layer drying kinetics of ghost chilli pepper (Capsicum Chinense Jacq.) dried in a forced convection solar tunnel dryer. *Renew. Energy* **105**, 583–589 (2017).

[75] Yang, L. et al. A new automatic sugarcane seed cutting machine based on internet of things technology and RGB color sensor. *PLoS ONE* **19**, e0301294 (2024). 38547096 10.1371/journal.pone.0301294 PMC10977673

[76] Singh, P. & Gaur, M. K. Environmental and economic analysis of novel hybrid active greenhouse solar dryer with evacuated tube solar collector. *Sustain. Energy Technol. Assess.* **47**, 101428 (2021).

[77] Khater, E.-S.G. et al. Assessment of a LPG hybrid solar dryer assisted with smart air circulation system for drying basil leaves. *Sci. Rep.* **14**, 23922 (2024). 39397051 10.1038/s41598-024-74751-4 PMC11471770

[78] Prakash, O. & Kumar, A. Environomical analysis and mathematical modelling for tomato flakes drying in a modified greenhouse dryer under active mode. *Int. J. Food Eng.* **10**, 669–681 (2014).

[79] Nayak, S., Naaz, Z., Yadav, P. & Chaudhary, R. Economic analysis of hybrid photovoltaic-thermal (PVT) integrated solar dryer. *Int. J. Eng. Invent.* **1**, 21–27 (2012).

[80] Vijayan, S., Arjunan, T. V. & Kumar, A. Exergo-environmental analysis of an indirect forced convection solar dryer for drying bitter gourd slices. *Renew. Energy* **146**, 2210–2223 (2020).

[81] Elshehawy, S. M. & Mosad, G. A. Mathematical modeling of tilapia fish fillets dried in thin layer. *J. Soil Sci. Agric. Eng.* **13**, 359–364 (2022).

[82] Haftom, Z., Tsegay, T. & Tesfay, M. Evaluation of solar dryers on drying and sensory properties of salted Tilapia filets, Tigray, Northern Ethiopia. *ISABB J. Food Agric. Sci.* **7**, 10–18 (2017).

[83] Fernando, W. J. N., Low, H. C. & Ahmad, A. L. Dependence of the effective diffusion coefficient of moisture with thickness and temperature in convective drying of sliced materials. A study on slices of banana, cassava and pumpkin. *J. Food Eng.* **102**, 310–316 (2011).

[84] Maskan, A., Kaya, S. & Maskan, M. Hot air and sun drying of grape leather (pestil). *J. Food Eng.* **54**, 81–88 (2002).

[85] Nguyen, M.-H. & Price, W. E. Air-drying of banana: Influence of experimental parameters, slab thickness, banana maturity and harvesting season. *J. Food Eng.* **79**, 200–207 (2007).

[86] Tütüncü, M. A. & Labuza, T. P. Effect of geometry on the effective moisture transfer diffusion coefficient. *J. Food Eng.* **30**, 433–447 (1996).

[87] Yan, Z., Sousa-Gallagher, M. J. & Oliveira, F. A. R. Shrinkage and porosity of banana, pineapple and mango slices during air-drying. *J. Food Eng.* **84**, 430–440 (2008).

[88] Guan, Z., Wang, X., Li, M. & Jiang, X. Mathematical modeling on hot air drying of thin layer fresh tilapia fillets. *Pol. J. Food Nutr. Sci.* **63**, 25–34 (2013).

[89] Jain, D. & Pathare, P. B. Study the drying kinetics of open sun drying of fish. *J. Food Eng.* **78**, 1315–1319 (2007).

[90] Boudhrioua, N., Djendoubi, N., Bellagha, S. & Kechaou, N. Study of moisture and salt transfers during salting of sardine fillets. *J. Food Eng.* **94**, 83–89 (2009).

[91] Asdrubali, F. et al. A review of structural, thermo-physical, acoustical, and environmental properties of wooden materials for building applications. *Build. Environ.* **114**, 307–332 (2017).

[92] Baird, G., Alcorn, A. & Haslam, P. The energy embodied in building materials-updated New Zealand coefficients and their significance. *Trans. Inst. Prof. Eng. N. Z. Civ. Eng. Sect.* **24**, 46–54 (1997).

[93] Prakash, O., Kumar, A. & Laguri, V. Performance of modified greenhouse dryer with thermal energy storage. *Energy Rep.* **2**, 155–162 (2016).

[94] Kumar Singh, A. Material conscious energy matrix and enviro-economic analysis of passive ETC solar still. *Mater. Today Proc.* **38**, 1–5 (2021).

[95] Charoentanaworakun, C. et al. Minimum carbon credit cost estimation for carbon geological storage in the Mae Moh Basin, Thailand. *Energies (Basel)* **17**, 2231 (2024).

